# ZNF131-BACH1 transcriptionally accelerates RAD51-dependent homologous recombination repair and therapy-resistance of non-small-lung cancer cells by preventing their degradation from CUL3

**DOI:** 10.7150/thno.97593

**Published:** 2024-10-28

**Authors:** Mingwei Fan, Quanbo Liu, Xiaowen Ma, Yufeng Jiang, Yilong Wang, Shuting Jia, Yingtong Nie, Ruoyi Deng, Pengchong Zhou, Shuyu Zhang, Siyu Jiang, Mengyao Guan, Yuekang Hou, Yuan Miao, Yong Zhang, Xiupeng Zhang

**Affiliations:** 1 Department of Pathology, College of Basic Medical Sciences and First Affiliated Hospital of China Medical University, Shenyang, China.; 2 Department of Pathology, The Second Affiliated Hospital of Shandong First Medical University, Shandong, China.; 3 Department of Respiratory Medicine, Shengjing Hospital of China Medical University, Shenyang, China.; 4 Second Department of Clinical Medicine, China Medical University, Shenyang, China.; 5 Department of Emergency, First Affiliated Hospital of China Medical University, Shenyang, China.; 6 Department of Radiation Oncology, First Affiliated Hospital of China Medical University, Shenyang, China.; 7 First Department of Clinical Medicine, China Medical University, Shenyang, China.; 8 Department of Pathology, Cancer Hospital of China Medical University, Liaoning Cancer Hospital and Institute, Shenyang, China.

**Keywords:** ZNF131, BACH1, RAD51, CUL3, Homologous recombination repair

## Abstract

**Rationale:** Both bulk RNA-sequencing and GEO database upon chemotherapy to non-small cell lung cancer (NSCLC) cells reveal that ZNF131 (Zinc Finger Protein 131) maybe a crucial transcriptional factor involved. However, it is a recently discovered protein with largely unexplored expression patterns and biological functions.

**Methods:** Bioinformatics analyses and immunohistochemistry staining were assessed to detect both mRNA and protein levels of ZNF131 in NSCLC specimens and cell lines. Next, colony formation assay, MTT assay, EdU assay, transwell assay, flow cytometric analysis, sphere formation assay, western blotting analysis, mouse xenograft model analysis, immunofluorescence assay, and reverse transcriptase-polymerase chain reaction were performed to investigate the effect of ZNF131 interaction on proliferation, invasion, stemness, chemotherapy sensitivity. RNA-sequencing assay, RNA-microarray, and ChIP-sequencing assay were used to identify candidate downstream target genes. Further, liquid chromatography-tandem mass spectrometry analysis, GST pull-down assay, and immunoprecipitation assays were performed to evaluate the interactions between ZNF131, BACH1, and CUL3.

**Results:** ZNF131 was elevated in NSCLC specimens and cell lines, which significantly correlates with advanced TNM stage and poor prognosis in NSCLC patients. ZNF131 overexpression promotes NSCLC cell proliferation, invasion, and stemness both *in vitro* and *in vivo*. ZNF131 appears to target the RAD51 gene within a well-defined region (-668bp to -403bp) of the RAD51 promoter. ZNF131 contributes to RAD51-dependent homologous recombination (HR), primarily through its Zinc Finger and BTB domains. ZNF131-BACH1 interaction, mediated by their respective BTB domains, enhances the stability of both proteins, effectively preventing their ubiquitin-mediated degradation by CUL3. The ZNF131-BACH1 partnership significantly amplifies RAD51-dependent HR, resulting in expedited resistance to both radiotherapy and chemotherapy in NSCLC patients. Desoxyrhaponticin was shown to halt NSCLC progression and orchestrate a synergistic effect together with chemotherapy at least partially by targeting ZNF131.

**Conclusions:** Our findings indicate that ZNF131 exhibits heightened expression in NSCLC, driving essential processes such as proliferation, invasion, and stemness by transcriptionally activating RAD51. The ZNF131-BACH1 interaction serves as a crucial enhancer, further boosting RAD51 transcription and ultimately accelerating therapy resistance in NSCLC.

## Introduction

Lung cancer remains the leading cause of mortality among all human malignancies, boasting a less than 15% 5-year overall survival rate 1. Non-small cell lung cancer (NSCLC) predominates, accounting for 85% of all diagnosed cases 2. While radiotherapy and chemotherapy continue to show promise in the treatment of NSCLC 3, persistent therapy resistance consistently hampers the prospects of improved prognosis. Consequently, there is an urgent imperative to elucidate more effective therapeutic targets that can enhance the sensitivity of lung cancer cells to radiotherapy and chemotherapy. Additionally, further research is needed to comprehensively understand the molecular mechanisms underlying therapy resistance in lung cancer.

Radiotherapy and chemotherapy induce DNA damage, leading to DNA double-strand breaks (DSBs), which subsequently trigger the demise of tumor cells 4. Two primary pathways, homologous recombination (HR) and non-homologous end joining (NHEJ), are recognized as the fundamental mechanisms for repairing these damaged cells 5. NHEJ operates continuously throughout the cell cycle, whereas HR predominates during the S and G2 phases 6. HR plays a pivotal role in maintaining genomic integrity and commences at DNA ends that feature 3′-overhanging single-stranded DNA (ssDNA). This initial step involves the binding of replication protein A (RPA) to the ssDNA 7. Following RPA binding, RAD51 displaces RPA from the ssDNA, forming the RAD51-ssDNA complex, which expedites the processing of the primary DNA strand invasion intermediate known as the D-loop 8. RAD51 levels increase during the progression of various malignancies 9 and play critical roles in conferring resistance to radiotherapy and chemotherapy 10. However, the underlying mechanisms governing the modulation of RAD51 expression and activity remain unclear.

The transcriptional program determines disparate biological processes and cell fates 11. We are aiming to explore pivotal transcriptional factors (TFs) involving therapy resistance of lung cancer by assessing an investigation strategy using bulk RNA-sequencing array as well as GEO databases. It revealed that 2 TFs (ZNF131 and SMARCB1) were identified. ZNF131 was chosen for further study, as it was demonstrated that ZNF131 plays a crucial role in maintaining stemness and promoting the proliferation of glioma cells in previous studies 12. ZNF131 belongs to the POK family and possesses a BTB/POZ domain in N-terminal and five Zinc Finger (ZF) domains in the C-terminal 13. While downstream targets of ZNF131 remain unidentified.

In this investigation, we employed RNA- and ChIP-sequencing arrays to explore the possibility that RAD51 might be a target gene subject to transcriptional activation by ZNF131. Furthermore, we elucidated the activation of the ZNF131-RAD51 axis in the context of resistance to both radiotherapy and chemotherapy.

## Results

### ZNF131 is a candidate transcriptional factor associated with chemotherapy resistance of NSCLC

**Figure 1:**
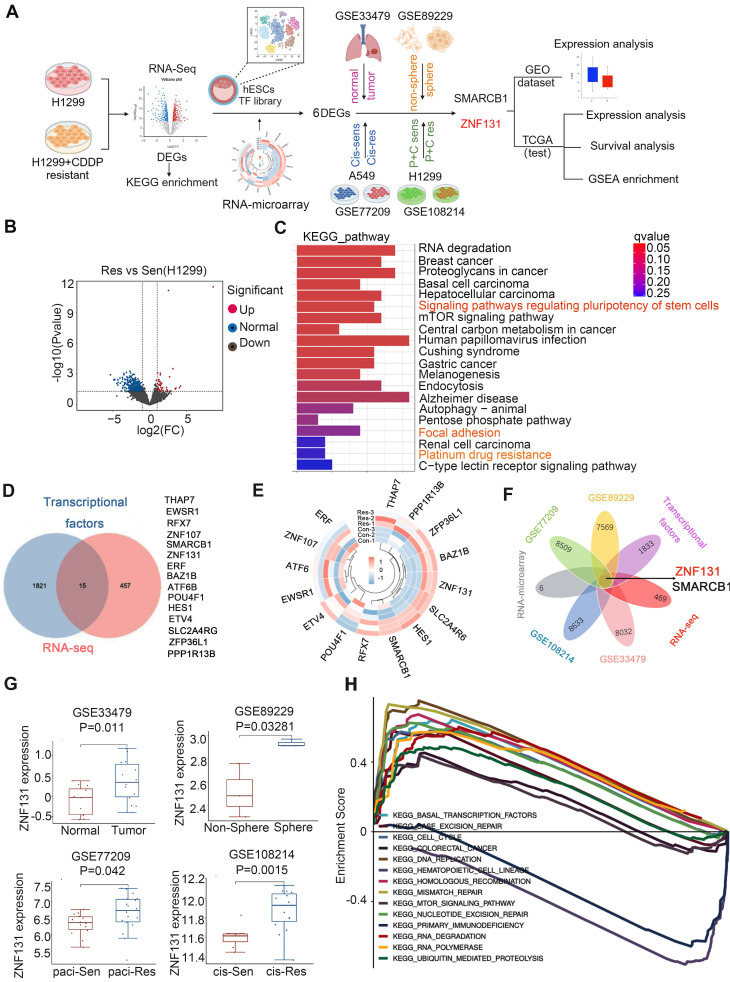
** Identification of ZNF131 as an impeller of chemotherapy for lung cancer.** (A) Flowchart for identification of ZNF131 as an impeller of chemotherapy of lung cancer. (B) Volcano plot of DEGs of RNA-sequencing between H1299- and H1299-CDDP-resistant cells. (C) KEGG pathway was performed according to the DEGs with RNA-sequencing data. (D) Venn analysis was performed between DEGs of RNA-sequencing data and published TF database to screen the overlap TFs during chemotherapy resistance of lung cancer. (E) Heat map of differential TFs after assessing RNA-microarray between H1299- and H1299-CDDP-resistant cells. (F) Venn analysis was further performed to identify TFs among RNA-sequencing, TF database, and 4 GEO databases (GSE33479, DEGs between non-cancerous and cancerous specimens; GSE89229, DEGs between non-sphere and sphere NSCLC cell line; GSE77209, DEGs between paclitaxel-sensitive- and resistant-H1299 cells; GSE108214, DEGs between cis-sensitive- and resistant-A549 cells). (G) ZNF131 mRNA expression was examined in four GEO databases. (H) GSEA was performed to detect the biological process and signaling pathway according to the DEGs upon high ZNF131 expression in NSCLC.

Initially, a bulk RNA sequencing array was performed on H1299 cells and their cisplatin-resistant counterparts to identify potential candidates involved in chemotherapy resistance in lung cancer. Subsequently, RNA microarray and Venn analysis were used to identify potential TFs based on a publishing TF database. The GEO database was utilized to validate the expression of candidate TFs (Figure 1A). RNA sequencing data revealed 54 upregulated and 418 downregulated genes associated with chemotherapy resistance. These differential expressed genes (DEGs) were enriched in pathways regulating stem cell pluripotency, focal adhesion, and platinum drug resistance (Figure 1B and C). Fifteen TFs were identified through Venn analysis comparing DEGs and the published TF database 11 (Figure 1D). RNA microarray was then conducted to examine the mRNA expression of these 15 TFs in H1299 and H1299 cisplatin-resistant cells, revealing six differentially expressed TFs, six of which were upregulated (Figure 1E). The expression of these six TFs was further analyzed using four GEO databases (GSE33479, GSE89229, GSE77209, GSE108214) to compare non-cancerous vs. cancerous samples, non-sphere vs. sphere NSCLC cell lines, paclitaxel-sensitive vs. resistant H1299 cells, and cisplatin-sensitive vs. resistant A549 cells. Venn analysis of differential TFs and DEGs across these four GEO datasets identified two key TFs, ZNF131 and SMARCB1 (Figure 1F). ZNF131 was selected for further investigation due to its critical role in maintaining stemness and promoting tumor cell proliferation 12, 14, 15. Additional analysis of ZNF131 expression across the four GEO datasets, along with Gene Set Enrichment Analysis (GSEA) revealed that DEGs with elevated ZNF131 expression were enriched in cell cycle regulation, homologous recombination, and DNA replication processes (Figure 1G and H).

### ZNF131 overexpression in NSCLC tissues and its correlation with poor prognosis

**Figure 2:**
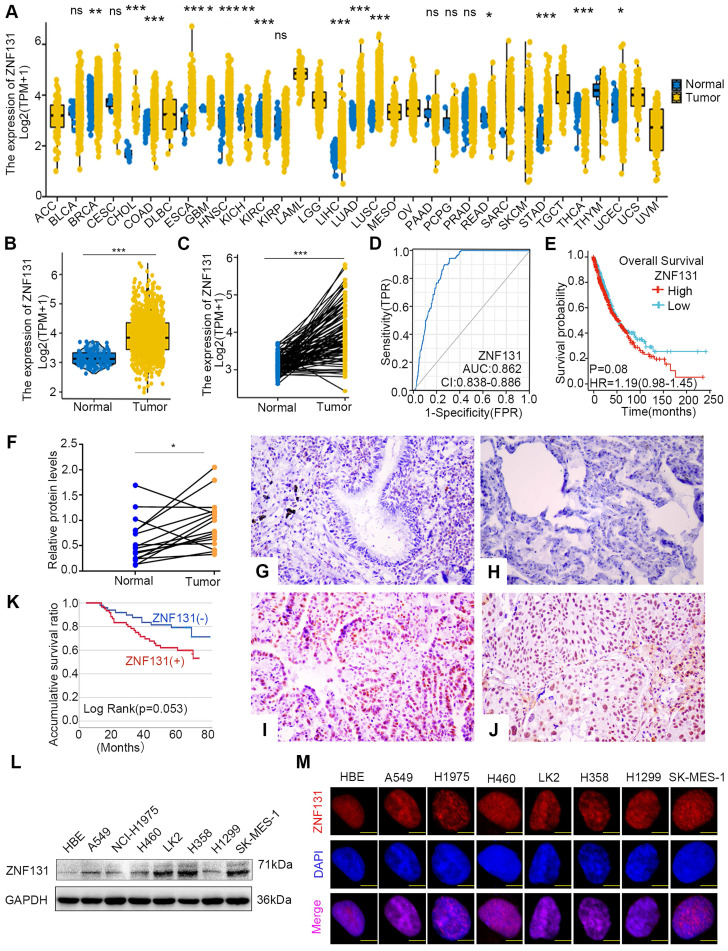
** ZNF131 is elevated in the nucleus of NSCLC specimens and cell lines.** (A) The mRNA expression of ZNF131 across various cancer types and normal tissues was investigated using the TCGA database. (B-C) Comparative analysis of ZNF131 mRNA levels between non-cancerous and cancerous tissues, utilizing TCGA data. (D) Construction of an Area under the Curve (AUC) curve based on ZNF131 expression in lung cancer. (E) Kaplan-Meier survival curves illustrate the correlation between ZNF131 mRNA expression and overall survival among lung cancer patients. (F) ZNF131 protein levels in freshly isolated samples from 16 lung cancer patients, were assessed through Western blotting. (G-H) Representative images of immunohistochemistry staining for ZNF131 in normal bronchial epithelial cells and normal alveolar epithelial cells. (I-J) Immunohistochemistry staining images of ZNF131 in adenocarcinoma and squamous cell carcinoma. (K) Kaplan-Meier survival curves demonstrating the association between ZNF131 protein expression and overall survival in lung cancer patients. (L-M) Evaluation of ZNF131 protein levels and subcellular localization through Western blotting and immunofluorescence assays, with a scale bar of 10 μm. Quantitative data represent the mean ± SD of three independent experiments. Statistical significance levels are denoted as * P < 0.05, ** P < 0.01, *** P < 0.001, based on t-test analysis.

Next, utilizing the data from The Cancer Genome Atlas (TCGA) database, we identified a substantial elevation of ZNF131 mRNA expression in cancerous tissues compared to non-cancerous counterparts (Figure 2A). In particular, a pronounced upregulation of ZNF131 expression in both paired and non-paired lung cancer specimens became evident (Figure 2B and C). Furthermore, the risk score curve and Kaplan-Meier analysis demonstrated that lung cancer patients with heightened ZNF131 expression exhibited significantly reduced survival times and rates in comparison to those with lower ZNF131 expression (Figure 2D and E). Subsequent immunohistochemistry (IHC) and western blotting assays staining confirmed the elevated nuclear expression of ZNF131 in tumors relative to normal tissues (Figure 2F-J and Figure S1). The positivity rate of ZNF131 in lung cancer tissues (51.4%, 56/109) was significantly higher than that in normal lung specimens (15.8%, 6/38, P < 0.001). Subsequent statistical analysis unveiled a significant positive correlation between ZNF131 expression and advanced TNM stage (P = 0.044) as well as positive lymph node metastasis (P = 0.02), while no significant associations were observed with age, sex, histology, tumor size, and differentiation (P > 0.05, Table 1). Kaplan-Meier analysis further indicated that NSCLC patients with higher ZNF131 expression (52.953 ± 3.204) experienced shorter overall survival times compared to those with lower ZNF131 expression (69.837 ± 2.982, P = 0.053, Figure 2K). Nevertheless, Cox univariate analysis did not identify positive ZNF131 expression as an independent prognostic factor in NSCLC patients (Table 2).

Further immunoblotting assay in seven NSCLC cell lines and one normal bronchial epithelial cell line (HBE) also identified higher ZNF131 expression in all tested NSCLC cell lines in comparison to HBE (Figure 2L). Moreover, in line with immunohistochemistry staining results, immunofluorescence assays showed that ZNF131 predominantly localized within the nucleus of NSCLC cells (Figure 2M).

### ZNF131 overexpression enhances NSCLC proliferation, invasion and stemness *in vitro* and *in vivo*

**Figure 3:**
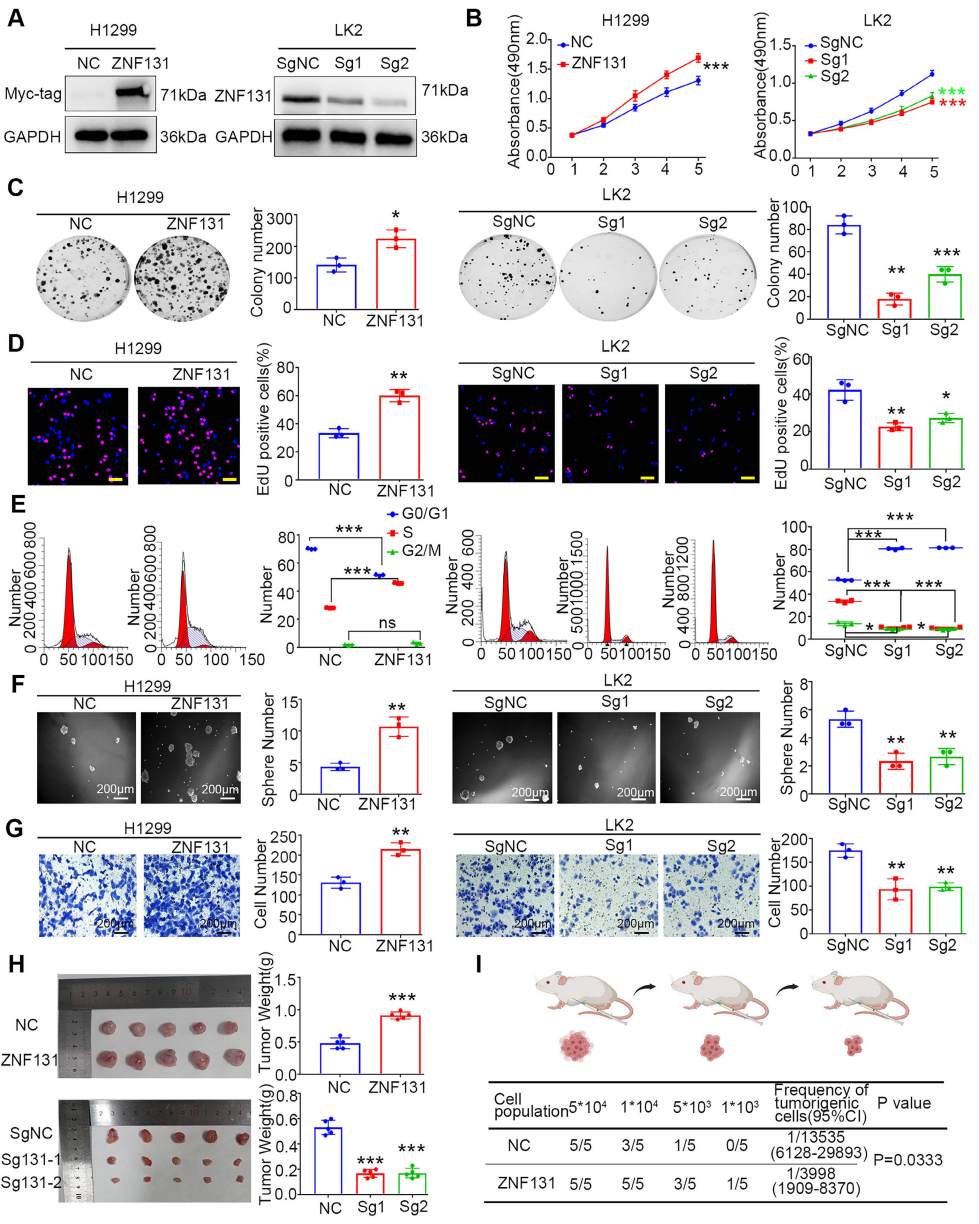
** ZNF131 Promotes Proliferation, Invasion, and Stemness of NSCLC Cells *In Vitro* and *In Vivo*.** (A) Western blotting confirmed the efficiency of ZNF131 overexpression in H1299 or A549 cells and ZNF131 knockout in LK2 or H358 cells. (B) MTT assays, (C) colony formation assays, (D) EdU assays (scale bar =100 μm), and (E) flow cytometry were conducted to assess the impact of ZNF131 overexpression and silencing on NSCLC cell proliferation. (F) Sphere formation assays and (G) transwell assays were performed to evaluate the influence of ZNF131 overexpression or deletion on stemness and invasion capabilities. (H) Xenografts and (I) limiting dilution xenografts were employed to examine the effects of ZNF131 ectopic expression and knockout on cell proliferation and stemness. Quantitative data are presented as mean ± SD from three independent experiments. Statistical significance levels are indicated as * P < 0.05, ** P < 0.01, *** P < 0.001, based on t-test analysis.

To gain insights into the biological processes influenced by heightened ZNF131 expression, we conducted Kyoto Encyclopedia of Genes and Genomes (KEGG) pathway and Gene Ontology (GO) analysis, as depicted in Figure S2A and B, respectively. Subsequently, we modulated ZNF131 expression levels by transfecting PCMV6-ZNF131-myc or deleting the gene using two distinct sgRNAs in H1299, A549, LK2, and H358 cells (Figure 3A and Figure S2C). We assessed the impact of these manipulations on NSCLC cells through a battery of assays, including MTT (Figure 3B and Figure S2D), colony formation (Figure 3C and Figure S2E), EdU (Figure 3D and Figure S2F), and flow cytometry (Figure 3E and Figure S2G).

These analyses unequivocally demonstrated that ZNF131 overexpression enhanced proliferation, while its depletion had the opposite effect. Furthermore, sphere formation assays and transwell assays revealed that ZNF131 overexpression promoted stemness and invasion in NSCLC cells, while its depletion attenuated this trait (Figure 3F-G and Figure S2H-I). *In vivo* xenograft assays reinforced the *in vitro* observations, showing that NSCLC cell proliferation was upregulated or downregulated upon ectopic ZNF131 expression or ZNF131 deletion (Figure 3H). To further explore the effects of ZNF131 on stemness *in vivo*, we conducted limiting dilution xenograft assays, which confirmed that ZNF131 overexpression elevated the stemness of NSCLC (Figure 3I).

### RAD51 identified as a downstream effector of ZNF131

**Figure 4:**
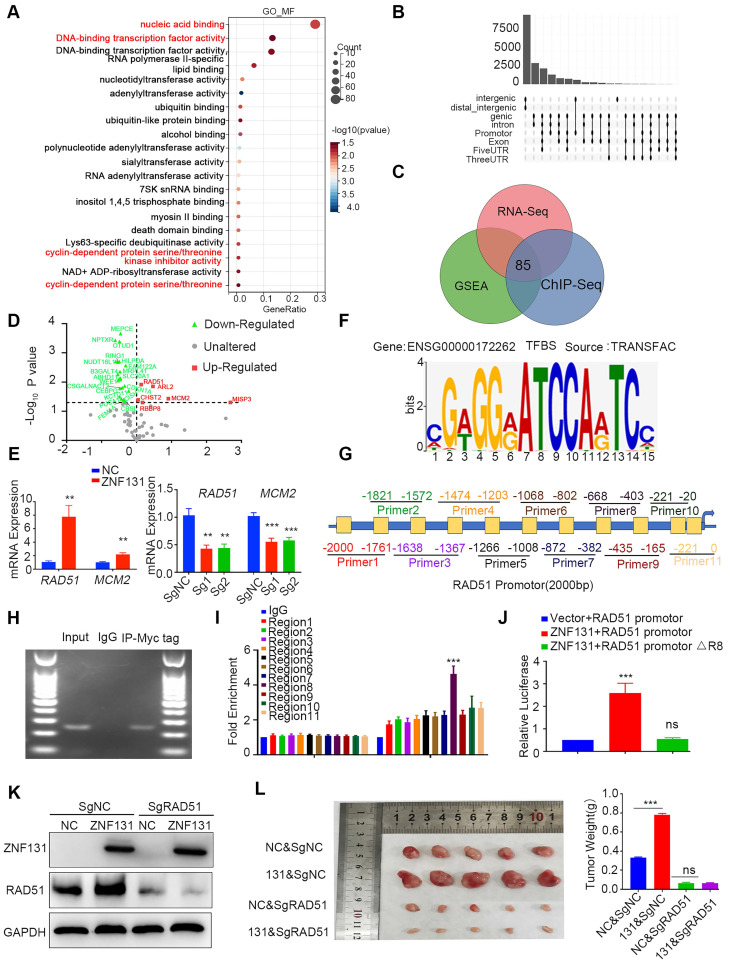
** Identification of RAD51 as a Downstream Target of ZNF131.** (A) GO analysis was conducted to identify the biological processes significantly associated with the overexpression of ZNF131 in NSCLC cells. (B) ChIP-sequencing was utilized to screen for candidate downstream factors that might be transcriptionally modulated by ZNF131. (C) Venn diagram analysis was performed to identify common elements among GSEA, RNA-seq, and ChIP-sequencing array analyses. (D) PCR-array was employed to investigate 85 DEGs after Venn analysis in response to ectopic ZNF131 expression in H1299 cells. (E) QPCR assay was employed to measure RAD51 and CDK1 mRNA levels following ZNF131 overexpression or knockout in NSCLC cells. (F) The TRANSFAC database was used to identify the binding site for ZNF131. (G) Diverse primers were designed for the examination of the binding site on the RAD51 promoter. (H) ChIP assay was performed to examine the interaction between the RAD51 promoter and ZNF131. (I) Further ChIP assays were performed to elucidate the precise binding region of ZNF131 on the RAD51 promoter. (J) We employed luciferase assays to explore the transcriptional effect of ZNF131 on RAD51 within the region spanning from -668 bp to -403 bp. (K) Western blotting was utilized to assess the expression of ZNF131 and RAD51 upon ZNF131 overexpression and RAD51 silencing on NSCLC cell lines. (L) Xenografts were used to evaluate the *in vivo* effect of ZNF131 on NSCLC proliferation. Quantitative data are presented as mean ± SD from three independent experiments. Statistical significance was determined using t-tests (** P < 0.01, *** P < 0.001).

We initiated an RNA sequencing analysis to identify DEGs and elucidate the biological processes associated with ZNF131 overexpression. A total of 669 upregulated and 1548 downregulated DEGs were identified Table S1, Figure S3A). GO analysis highlighted the involvement of these DEGs in key processes, including nucleic acid binding, DNA-binding transcription factor activity, and cyclin-dependent protein serine/threonine kinase inhibitor activity (Figure 4A), consistent with findings from GSEA (Figure S2A-B). Given ZNF131's role as a transcriptional factor (16, we next performed Chromatin Immunoprecipitation sequencing (ChIP-seq) to validate key candidates Figure S3A-B). We identified 8410 regions (≤ 2kb) bound by ZNF131 (Figure 4B). Integrating RNA-seq, GSEA, and ChIP-seq data via Venn diagram analysis led to the identification of 85 genes (Figure 4C). GO analysis of these DEGs revealed enrichment in cell cycle and DNA replication processes (Figure S3C). To further refine our findings, RNA microarray analysis identified 22 downregulated and 6 upregulated genes, including MCM2 and RAD51 (Figure 4D). Analysis of the GEPIA database showed a significant positive correlation between ZNF131 and the mRNA levels of RAD51 and MCM2 (Figure S4A). Subsequent qPCR assays confirmed the upregulation of RAD51 and MCM2 upon ZNF131 overexpression and their downregulation upon ZNF131 depletion (Figure 4E).

We then explored potential ZNF131 binding sites using the TRANSFAC database (Figure 4F). RAD51 was selected for further investigation due to the presence of a putative ZNF131 binding sequence within its promoter, unlike MCM2. A luciferase reporter assay showed increased RAD51 transcription upon ZNF131 overexpression. To precisely locate the ZNF131 binding site on the RAD51 promoter, we designed several primers (Figure 4G). ChIP and qPCR assays pinpointed region 8 (-668 bp to -403 bp) as the crucial ZNF131 binding site (Figure 4H-I). Deletion of this region abolished the transcriptional enhancement induced by ZNF131 overexpression compared to the intact RAD51 promoter (Figure 4J).

To investigate the functional role of RAD51, we utilized RAD51 sgRNA or its specific inhibitor B02 to suppress RAD51 expression or function. Co-transfection of ZNF131 with RAD51 sgRNA, or the addition of B02, effectively nullified ZNF131-induced increases in proliferation, invasion, and stemness (Figure 4K and Figure S4B-E). Additionally, we assessed homologous recombination using the DNA damage marker γ-H2AX. ZNF131 overexpression reduced γ-H2AX foci formation, an effect reversed by co-transfection with RAD51 sgRNA or B02 treatment (Figure S4F). *In vivo* xenograft assays further reinforced our findings, indicating that RAD51 deletion could counterbalance the heightened proliferation of NSCLC cells induced by ZNF131 (Figure 4L).

### ZNF131 overexpression transcriptionally upregulates RAD51 dependent on both zinc finger and BTB domains

**Figure 5:**
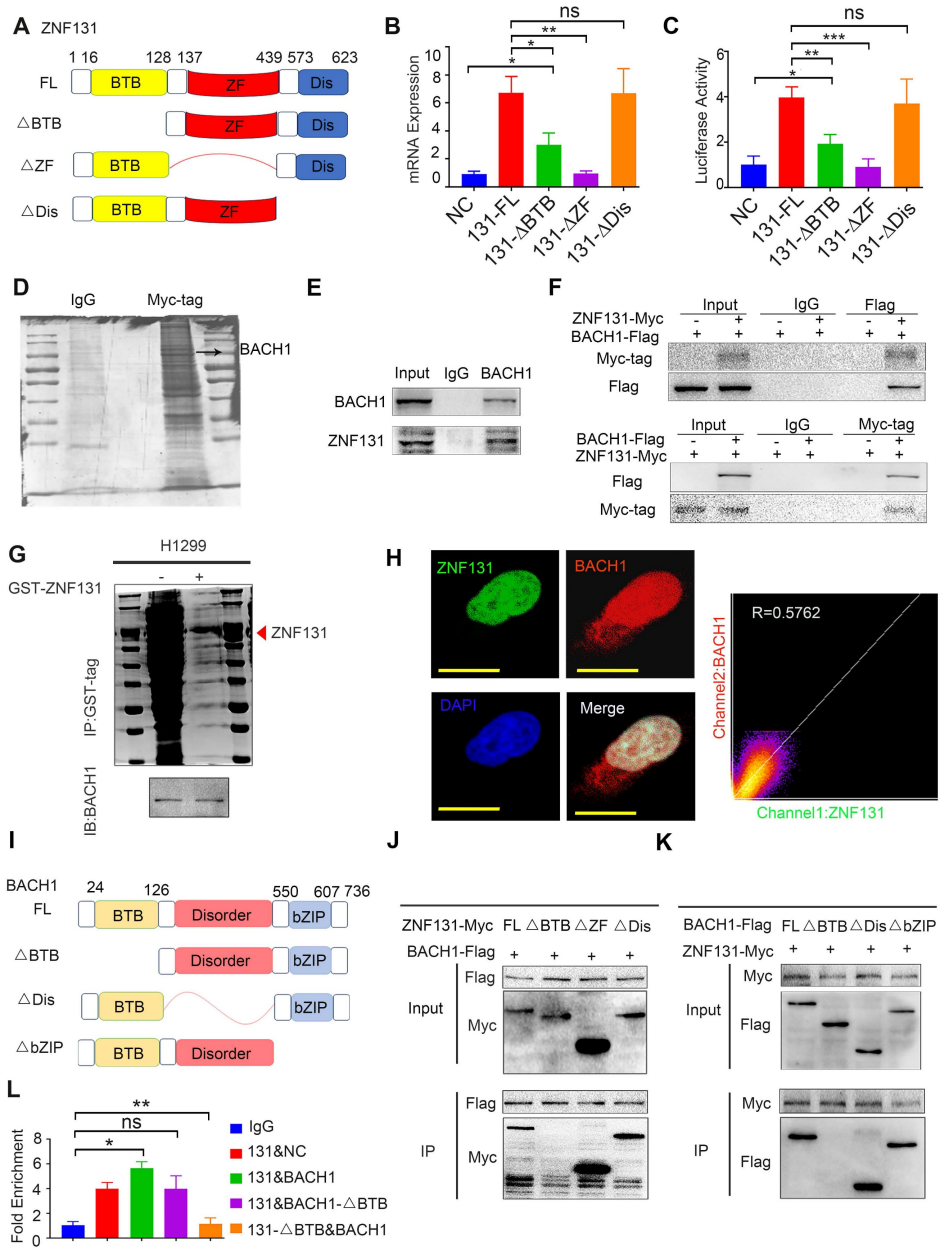
** Modulation of RAD51 by ZNF131 through ZF and BTB Domains.** (A) Divergent splicing mutant plasmids of ZNF131 were designed to examine the domain responsible for the upregulation of RAD51 transcription. The qPCR (B) and luciferase assays (C) were used to assess the mRNA expression and transcription upon overexpression of ZNF131-FL, ZNF131-∆ZF, ZNF131-∆BTB, and a control plasmid. (D) MS analysis was performed to identify potential interacting candidates with ZNF131. Both endogenous (E) and exogenous co-IP assay(F) were conducted to detect the interaction between ZNF131 and BACH1. (G) GST pull-down assay was used to confirm the direct binding between BACH1 and ZNF131. (H) An immunofluorescence assay was utilized to illustrate the co-localization of ZNF131 and BACH1. The subcellular localization coefficient of the ZNF131-BACH1 interaction was quantified using Fiji software (scale bar = 20 μm). (I) Divergent BACH1 splicing mutant plasmids were designed to elucidate the domain responsible for the interaction between ZNF131 and BACH1. (J-K) Co-IP assays were employed to pinpoint the specific domains responsible for binding between ZNF131 and BACH1 (L) ChIP assays were used to examine the transcription of RAD51 after overexpressing ZNF131 and various BACH1 splicing mutant plasmids. Quantitative data are presented as mean ± SD of three independent experiments. Statistical significance was determined by t-tests (* P < 0.05, ** P < 0.01, *** P < 0.001).

To delineate the precise domains responsible for the ZNF131-RAD51 axis, we generated diverse splicing mutant plasmids (Figure 5A). Per previous studies (16, we observed that the deletion of the Zinc Finger domain abolished the transcriptional activation and mRNA expression of RAD51 (Figure 5B-C). Interestingly, the deletion of the BTB domain of ZNF131 also partially abrogated RAD51 (Figure 5B-C), suggesting that the BTB domain may play a crucial role in modulating the function of ZNF131.

We performed mass spectrometry (MS) to identify potential binding proteins interacting with ZNF131, and we found 1296 proteins as potential binding partners, as illustrated in Figure 5D and detailed in Table S2. These proteins are known to form heterodimers through their BTB domains 17. BACH1, a BTB-domain-containing protein that has been demonstrated to modulate RAD51 18, was selected for further investigation. Subsequent Co-immunoprecipitation (Co-IP) assays revealed that both endogenous and exogenous ZNF131 interacted with BACH1 (Figure 5E-F). Moreover, a GST pull-down assay confirmed that ZNF131 directly binds to BACH1 (Figure 5G). Immunofluorescence assays indicated that ZNF131 co-localized with BACH1 in the nucleus of NSCLC cells, and this interaction was quantified using ImageJ software (Figure 5H).

To pinpoint the exact domains responsible for the ZNF131-BACH1 interaction, we designed divergent ZNF131 and BACH1 splice-mutant plasmids (Figure 5I). Co-IP assays revealed that the BTB domain in both proteins predominated in the ZNF131-BACH1 interaction (Figure 5J-K). Furthermore, it was observed that the co-overexpression of ZNF131 and BACH1, rather than ZNF131 + BACH1-∆BTB or ZNF131-∆BTB + BACH1, enhanced the transcription of RAD51 (Figure 5L).

### The mutual stabilization between ZNF131 and BACH1 preventing ubiquitin-dependent degradation initiated by CUL3

**Figure 6:**
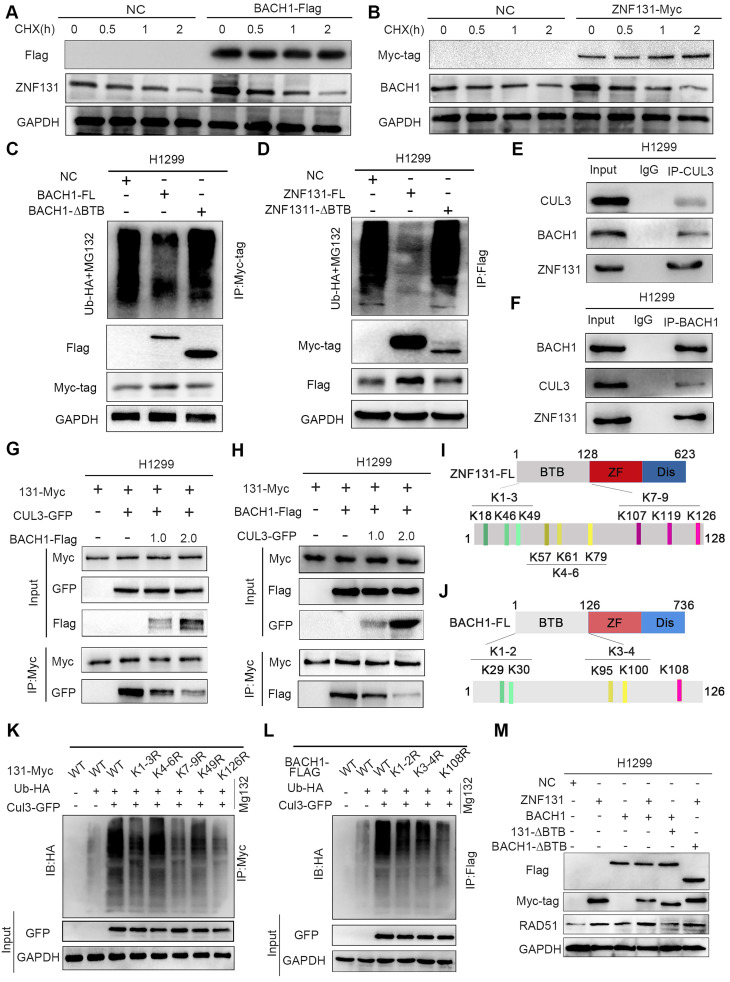
** Stabilization of ZNF131 and BACH1 by Preventing Ubiquitin-Mediated Degradation via CUL3.** (A-B) Following treatment with CHX at indicated time points, the expression of ZNF131 or BACH1 was evaluated by western blotting after overexpressing ZNF131 or BACH1. (C-D) The ubiquitination level of BACH1 or ZNF131 was determined using western blotting after transfected with ZNF131, ZNF131-∆BTB, or BACH1, BACH1-∆BTB, and control plasmids. (E-F) Endogenous co-IP assay was performed to detect the interaction between ZNF131, BACH1, and ZNF131 (G-H) Co-IP assay was used to evaluate the interaction among ZNF131, BACH1, and CUL3 following overexpressing BACH1 and CUL3 at different doses. (I-J) Divergent ZNF131 or BACH1 point-mutant plasmids were designed to elucidate the detailed lysines responsible for ubiquitin-mediated degradation of ZNF131 or BACH1 by CUL3. (K-L) The ubiquitination level of BACH1 or ZNF131 was determined using western blotting after transfected with diverse point-mutant plasmids of ZNF131 or BACH1 and control plasmids.(M) Immunoblotting analysis of Myc-tag, Flag-tag, RAD51 and GAPDH after transfection with ZNF131-myc, ZNF131-∆BTB-myc, BACH1-flag, BACH1-∆BTB-flag alone, or combinations of ZNF131-myc + BACH1-flag, ZNF131-∆BTB-myc + BACH1-flag, or ZNF131-myc + BACH1-∆BTB-flag, respectively.

Our subsequent objective is to investigate how the ZNF131-BACH1 interaction may accelerate the transcription of RAD51. We conducted an examination of both the mRNA and protein levels of ZNF131 and BACH1 following their respective overexpression. Interestingly, while mRNA levels remained unchanged, we observed a significant increase in protein levels for both ZNF131 and BACH1 upon overexpression Figure S5A-D). To further elucidate the underlying mechanisms, we treated cells with cycloheximide (CHX) to inhibit de novo protein synthesis of ZNF131 and BACH1. These experiments indicated that ZNF131 and BACH1 might impede each other's degradation (Figure 6A-B).

Our analysis, incorporating GSEA and RNA-seq, pointed to the involvement of ubiquitin-mediated degradation pathways (Figure S5E and Figure 4A). Additionally, GO analysis of ZNF131-binding candidates identified through MS assays confirmed their association with ubiquitin-mediated degradation (Figure S5F). Ubiquitination assays on ZNF131 and BACH1 following overexpression revealed that both proteins were protected from ubiquitin-dependent degradation, unlike their truncated forms, ZNF131-∆BTB or BACH1-∆BTB (Figure 6C-D). To identify the E3 ubiquitin ligase responsible for this process, we screened potential binding partners using MS, identifying CUL3 as a candidate. CUL3 is known to degrade BTB-domain-containing proteins (19. Further analysis predicted CUL3 as a key binding partner for both ZNF131 and BACH1 (www.thebiogrid.org, Figure S5G-H).

We identified a ternary complex involving ZNF131, BACH1, and CUL3 (Figure 6E-F). Importantly, CUL3 overexpression, in a dose-dependent manner, disrupted the interaction between ZNF131 and BACH1 (Figure. 6G-H). To pinpoint the specific lysine residues responsible for ubiquitin-mediated degradation by CUL3, we designed point mutations in ZNF131 and BACH1. Subsequent co-IP assays revealed that Lys126 in ZNF131 and Lys108 in BACH1 are likely the key residues targeted for degradation by CUL3 (Figure 6I-L). Accordingly, co-expressing ZNF131 and BACH1, but not their truncated forms (ZNF131 + BACH1-∆BTB or ZNF131-∆BTB + BACH1), synergistically increased both mRNA and protein expression of RAD51 (Figure 6M and Figure S5I). Our results suggest a competitive binding mechanism among ZNF131, BACH1, and CUL3 that prevents the degradation of ZNF131 or BACH1, ultimately promoting RAD51 transcription.

### ZNF131 overexpression accelerates radiation or chemotherapy resistance

**Figure 7:**
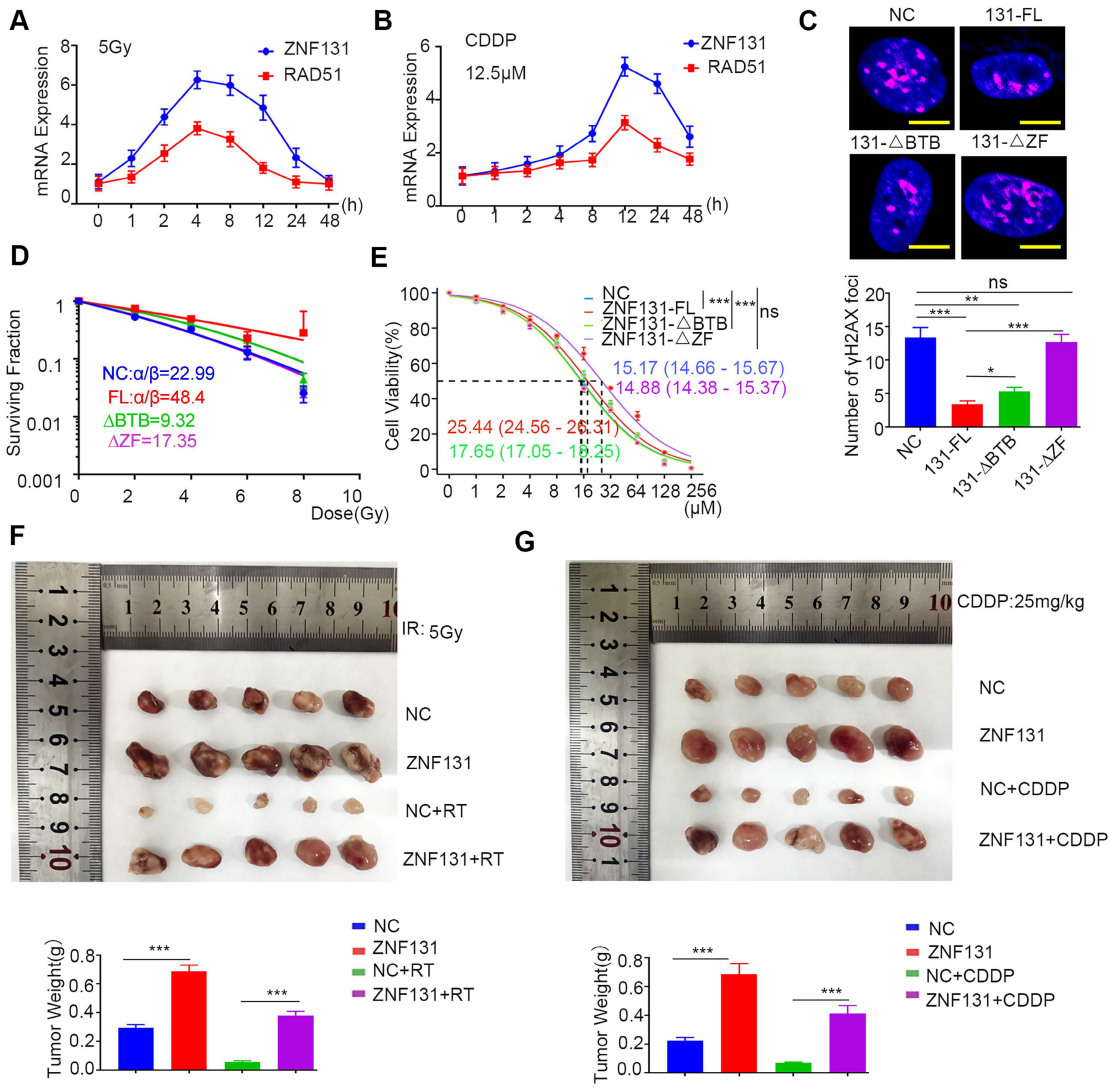
** Accelerated Resistance to Radiation and Chemotherapy upon ZNF131 Overexpression.** (A-B) QPCR assays were conducted to evaluate the mRNA expression of ZNF131 and RAD51 after treating NSCLC cells exposed to radiation (5Gy) and CDDP (12.5μM) at various time points. (C) representative immunofluorescence images of the number of γ-H2AX foci after overexpressing ZNF131-FL, ZNF131-∆ZF, ZNF131-∆BTB, and control plasmids (scale bar =10μm) (D) Clonogenic cell survival curves were generated using the linear-quadratic model to assess the impact of ZNF131-FL, ZNF131-∆ZF, ZNF131-∆BTB, and control plasmids in combination with irradiation. (E) Determination of IC50 values in H1299 cells overexpressing ZNF131-FL, ZNF131-∆ZF, ZNF131-∆BTB, and control plasmids following treatment with CDDP. (F-G) Xenografts assays were performed to assess the effects of ZNF131 overexpression in combination with radiation or CDDP treatment. Quantification data are presented as mean ± SD of three independent experiments (two-sided t-test, ** P < 0.01, *** P < 0.001).

Homologous recombination, a RAD51-dependent process, plays a critical role in conferring therapy resistance to NSCLC 20, 21. Thus, our primary objective was to ascertain whether ZNF131 overexpression could promote therapy resistance in NSCLC cells. To achieve this, we exposed NSCLC cells to both radiation and cisplatin, monitoring the mRNA expression of ZNF131 and RAD51 at various time points. Our results demonstrated that the mRNA expression of both proteins peaked simultaneously, consistent with our prior findings (Figure 7A-B). Furthermore, we assessed the formation of γ-H2AX foci in cells overexpressing ZNF131-FL, ZNF131-∆ZF, ZNF131-∆BTB, and the control group after radiation and cisplatin treatment (Figure 7C). Subsequently, we conducted an analysis of the survival fractions of NSCLC cells after radiation treatment, revealing that ZNF131 overexpression could potentially accelerate resistance to radiation therapy compared to ZNF131-∆ZF and ZNF131-∆BTB (NC, α/β: 22.986 ± 15.908; ZNF131-FL, α/β: 48.398 ± 211.611; ZNF131-∆BTB, α/β: 9.320 ± 4.290; ZNF131-∆ZF, α/β: 17.345 ± 8.778; Figure 7D, Table 3). Additionally, the IC50 values for NSCLC cells treated with cisplatin increased upon ZNF131 overexpression, as opposed to overexpression of ZNF131-∆ZF and ZNF131-∆BTB (Figure 7E). Moreover, xenograft assays provided further evidence that ZNF131 overexpression, in contrast to ZNF131-∆ZF and ZNF131-∆BTB overexpression, accelerated resistance to radiation and cisplatin treatments (Figure 7F-G).

### Desoxyrhaponticin inhibition of ZNF131 abrogates NSCLC progression and sensitizes chemotherapy

**Figure 8:**
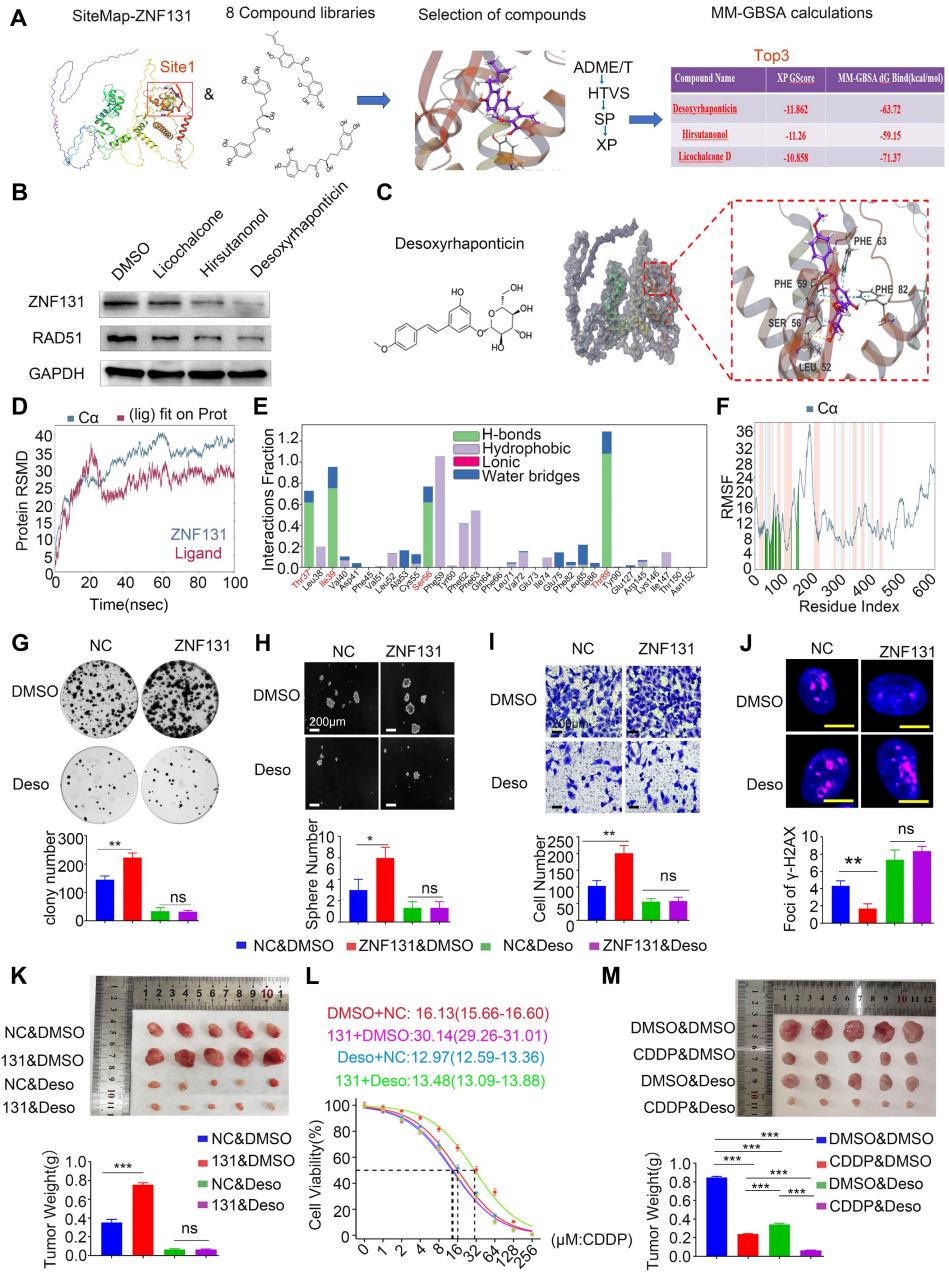
** Desoxyrhaponticin inhibited ZNF131 and abrogated NSCLC progression and sensitized chemotherapy.** (A) Typical workflow of docking-based virtual screening of FDA-approved drugs in 8 compound libraries (B) Western blotting assay was assessed to detect the expression of ZNF131 after treating NSCLC cells with Licochalcone, Hirsutanonol, and Desoxyrhaponticin (C) Molecular docking diagram of ZNF131 and Desoxyrhaponticin. (D) The RMSD plots for Desoxyrhaponticin; the blue color represents protein backbone fluctuations, and the red color represents ligand fluctuations. (E) The protein-ligand contacts show the bonding interactions fraction. (F) RMSF plots throughout the MD simulation. (G) Colony formation assays, (H) sphere formation assays, (I) transwell assays, and (J) IF assays were conducted to assess the impact on proliferation, stemness, invasion, and DNA damage of cells transfected with control vector or ZNF131-overexpressing vector treated with or without Desoxyrhaponticin. Scale bar = 200um (for Sphere/Transwell assay), Scale bar = 10um for IF assay. (K) Xenograft assays were performed to assess the effects of ZNF131 overexpression in combination with Desoxyrhaponticin. (L) Determination of IC50 values in cells overexpressing ZNF131 and treated with Desoxyrhaponticin. (M) Xenograft assays were performed to assess the effects in combination with CDDP and Desoxyrhaponticin treatment. Quantification data are presented as mean ± SD of three independent experiments (two-sided t-test, ** P < 0.01, *** P < 0.001).

Next, we assessed 8 compound libraries to identify a small molecular inhibitor that could specifically target ZNF131. The first step was high flux screening (HTVS), in which 38% of the compounds were retained. The second step was standard precision (SP), which included 47% of the first step. The third step was extra precision (XP), in which 18% of the compounds in the second step were retained. The screening process of these drugs is shown in Figure 8A. The top three screened drugs with high scores were selected (Desoxyrhaponticin, Hirsutanonol, and Licochalcone). Desoxyrhaponticin (Deso) revealed the most obvious inhibitory effect on ZNF131 (Figure 8B). Its chemical formula and docking mode with ZNF131 are shown in Figure 8C. The Root Mean Square Deviations (RFSP) plot analysis of the structures created during the Molecular Dynamics (MD) simulation illustrated stable binding between ZNF131 and Deso (Figure 8D). The interaction fraction was also monitored for the investigated complexes. For the complex, H-bond interaction was revealed as the primary contact between Deso and the residues of THR37, ILE39, SER56, and THR89 (Figure 8E and Figure S6A). The Root Mean Square Fluctuation (RMSF) was used to characterize local changes along the protein chain. We found RMSF curve fluctuated dramatically around the residue index 100 to 200, reflecting that the region was flexible (Figure 8F). The ligand torsions plot summarizes the conformational evolution of every rotatable bond in the ligand throughout the simulation trajectory. Deso and ZNF131 have only one chemical bond (blue) that can be reversed, which is more stable than the other two compounds Figure S6B-C). MD simulation data of the other two drugs are in Figure S7A-F and S8A-F. Deso also abrogated the elevated proliferation, invasion, stemness, and DNA damage induced by overexpression of ZNF131 *in vitro* (Fig 8G-J). Xenograft assays indicated that Deso may block elevated proliferation caused by ZNF131 overexpression and sensitize cisplatin treatment of lung cancer cells *in vitro* and *in vivo* (Figure 8K-M).

### ZNF131 expression positively correlated with neoadjuvant chemotherapy- and radiation-resistance in human NSCLC samples

**Figure 9:**
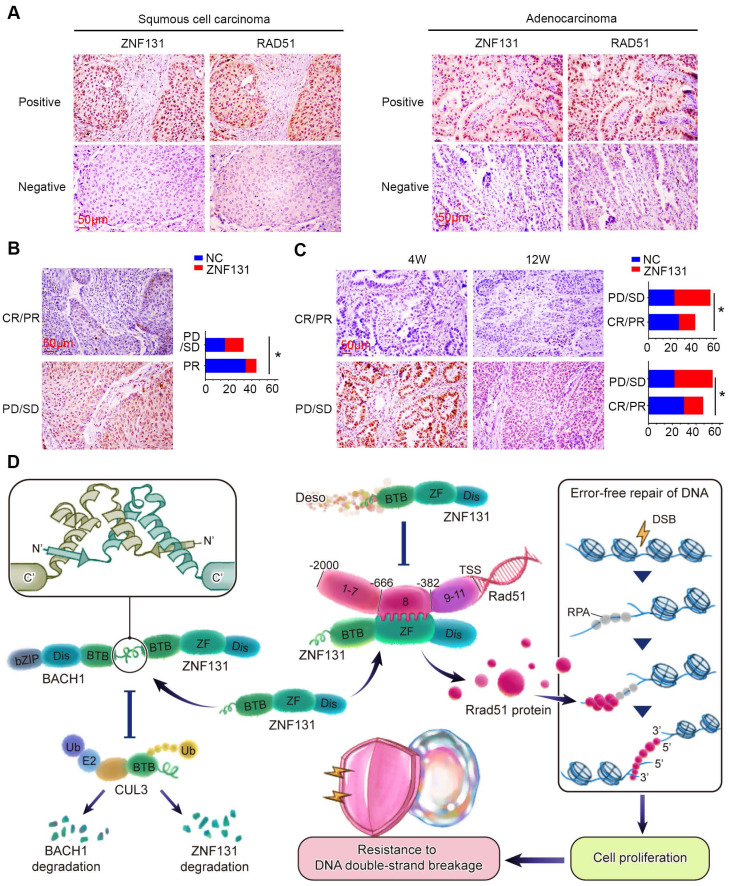
** Positive Correlation Between ZNF131 Expression, RAD51, and Chemotherapy/Radiotherapy Resistance in NSCLC Specimens.** (A) Representative images of immunohistochemistry staining images of ZNF131 and RAD51 in human NSCLC specimens. (B) Representative immunohistochemistry staining images of ZNF131 in specimens from lung cancer patients with varying therapeutic responses (Partial Response/Complete Response or Progressive Disease/Stable Disease). (C) Quantitative data, analyzed using Spearman's rank correlation, reveal the association between ZNF131 expression and radiotherapy responses at both 4 weeks (left panel) and 12 weeks (right panel) (D) Pathway diagram for ZNF131 functional activity interaction with BACH1 in NSCLC cells. Quantitative results are presented as Mean ± SD from three independent experiments (t-test, * P < 0.05).

We performed IHC staining to explore the relationship between ZNF131 expression and its downstream factor, RAD51, in human NSCLC samples. Our analysis revealed a significant and positive correlation between ZNF131 and RAD51 expression (P < 0.001; Figure 9A, Table 4. Our IHC staining results also indicated that ZNF131 expression was significantly lower in patients who were sensitive to chemotherapy treatment compared to those who exhibited resistance (P = 0.014, Figure 9B).To further examine the link between ZNF131 expression and response to radiation therapy, we evaluated the association between ZNF131 expression and the treatment response in the same patient cohort. We observed that both ZNF131 and RAD51 expression were significantly associated with a poor overall response rate (ORR). Four weeks after irradiation, patients with positive ZNF131 expression had an ORR of 31.3%, whereas those with negative ZNF131 expression had an ORR of 53.8% (P = 0.027). Similarly, twelve weeks after irradiation, patients with positive ZNF131 expression had an ORR of 33.3%, whereas those with negative ZNF131 expression had an ORR of 57.5% (P = 0.017, Figure 9C). In summary, our study unveiled that ZNF131 was highly expressed in the nucleus of lung cancer cells, and this expression was positively correlated with advanced TNM stage, lymph node metastasis, and poor prognosis. ZNF131 overexpression enhanced NSCLC proliferation, invasion, and stemness by transcriptionally activating RAD51 through its ZF domain. Additionally, ZNF131 interacted with BACH1 via their BTB domain and stabilized each other, potentially preventing ubiquitin-dependent degradation by CUL3. The ZNF131-RAD51 axis was found to be activated during NSCLC resistance to radiation and chemotherapy, which could be halted by Desoxyrhaponticin (Figure 9D).

## Discussion

Our investigations revealed that ZNF131 exhibited pronounced nuclear expression in both NSCLC specimens and cell lines. Importantly, this heightened expression significantly correlated with advanced TNM stage, lymph node metastasis, and an unfavorable prognosis. To shed light on the mechanisms driving ZNF131 upregulation in NSCLC, we initially conducted an assessment using the cBioPortal database (http://www.cbioportal.org/). This analysis revealed that 10.12% (49/484) of squamous cell carcinoma cases and 7.73% (51/660) of adenocarcinoma cases exhibited gene amplification of ZNF131. However, the incidence of ZNF131 gene mutations was notably lower, standing at 0.62% (3/484) and 1.36% (9/660), respectively. We also investigated potential epigenetic modifications using the Cistrome database(http://cistrome.org/db/#/), which suggested the involvement of H3K4me3. Prior research has demonstrated the regulatory role of H3K4me3 in gene expression 22-25, raising the question of whether elevated ZNF131 levels result from epigenetic modifications by H3K4me3. Furthermore, we probed the m6A methylation status of ZNF131 (whistleepitranscriptome.com/), revealing the absence of m6A methylation sites. This observation suggests that the mRNA stability of ZNF131 might remain unaffected by pre-translational modifications.

Both ChIP-seq and RNA-seq assays provided evidence suggesting that RAD51 could be downstream of ZNF131, a finding further confirmed by RNA microarray analysis. The TRANSFAC database also supported this conclusion by indicating that RAD51 possesses a target sequence recognized by ZNF131. Notably, our analyses did not identify other predicted targets, such as MCM2 and CDKN1A, from the aforementioned assays 16. An intriguing observation emerged from our ChIP assay results, which revealed that region 8 (-668 bp to -403 bp) is responsible for ZNF131-mediated transcriptional activation. This region coincides with a palindromic sequence zone. It's worth noting that BTB-domain-containing proteins are known to form heterodimers, relying on their BTB domains 26, 27. Previously, Nickett S. Donaldson and his colleagues demonstrated that Kaiso, another BTB domain-containing transcription factor, could suppress the activity of ZNF131 by binding to its BTB domain. However, our MS assay results contradicted this notion by revealing that Kaiso was not a potential interaction candidate of ZNF131, consistent with the findings of Varier RA *et al.* 28. Instead, BACH1 emerged as an identified candidate from the MS assay, a protein also known to modulate homologous recombination and RAD51. Izhar *et al.* reported that over 70% of randomly tested transcription factors, including many with ZNF motifs, are localized to sites of DNA damage, with approximately 90% of this localization being PARP-dependent 29. Furthermore, they also indicated that the localization to damaged chromatin is dependent on DNA-binding domains and that fragments with only a ZnF motif but lacking an RRM domain showed impaired localization. ZNF131 is a ZNF protein with a ZnF motif, which lacks an RRM domain. Therefore, we hypothesize that ZNF131 may not exhibit the same damaged chromatin localization behavior as other ZNF proteins reported in that literature. Additionally, our various omics data did not provide evidence of any interaction between ZNF131 and PARP. Our results suggested that the interaction between BACH1 and ZNF131 might enhance RAD51 transcription rather than abrogate it. Collectively, our findings, in conjunction with those from previous studies 16, 30, suggest that the BTB domain may play a role in modulating transcriptional activity. However, the direction of transcriptional regulation, whether up-regulation or down-regulation, appears to depend on the specific binding partner involved.

MCM2 was also identified as a differentially expressed gene through ChIP-seq, RNA-seq, and subsequent RNA microarray analyses, and has been demonstrated to be crucial in DNA replication 31-34. However, our ChIP-seq data suggest that ZNF131 does not bind to the promoter of MCM2. Instead, we focused on RAD51 for further study. Interestingly, many transcriptional factors can bind to intragenic or even coding sequences (CDS) to exert regulatory effects 35-37. Therefore, we sought to determine whether the increased proliferation and stemness observed upon ZNF131 overexpression might be partly mediated through MCM2. To test this, we co-transfected H1299 cells with MCM2 siRNA and a ZNF131 overexpression plasmid. The enhanced proliferation and stemness were at least partially blocked by MCM2 knockdown, suggesting that ZNF131 may exert its biological effects partly through the modulation of MCM2 Figure S9A-D). However, subsequent ChIP and luciferase assays confirmed that ZNF131 does not bind to the promoter of MCM2, which is consisted with our ChIP-seq data (Figure S9E-F). Although our ChIP-seq data indicated a potential interaction between ZNF131 and the ATM promoter, we did not further assess this interaction with a ChIP assay. Based on our current data, we speculate that ZNF131-induced resistance to radiotherapy and chemotherapy may primarily depend on RAD51. Future studies should further explore the role of DNA damage pathways in the context. CDKN1A was also identified in the Venn analysis, but it was not prioritized for further investigation for several reasons: 1. Both RAD51 and CDKN1A were shown as DEGs in the cell cycle, but DNA replication was also enriched, and MCM2 has a well-established role in DNA replication. 2. CDKN1A is a downstream effector of P53, and while A549 cell (used in our study) have wild-type P53, H1299 cell are P53-null. Since proliferation was enhanced in both cell lines upon ZNF131 overexpression, it is unlikely that the P53 signaling axis is involved. 3. CDKN1A can also be regulated independently of P53, a mechanism that may warrant investigation in further studies.

BACH1, recognized as a transcription factor, exhibited the ability to enhance RAD51's transcriptional activity when overexpressed. However, our ChIP assay results did not indicate direct binding of BACH1 to the RAD51 promoter (data not shown). To further validate this hypothesis, we co-transfected BACH1 and ZNF131 sgRNA, the abolishment of RAD51 upon deletion of ZNF131 could not be blocked by overexpressing BACH1. However, the protein could be at least partially rescued by overexpressing BACH1 (Figure S10A-B). Consistent with previous results, abrgation of ZNF131 might also suppress the expression of BACH1.This suggests that BACH1 might not directly activate RAD51 transcription but rather accelerates the process by binding with ZNF131.

ZNF131-BACH1 may stabilize each other by preventing ubiquitin-mediated degradation by CUL3. BACH1 has been proven to paly multifaceted roles in affecting diverse biological processes in malignancy, sometimes even with reverse effects on the same phenotypes or target genes (38, 39. Moreover, its phenotypic effects and genetic regulations might be modulated in an expression-level-dependent manner 40. Our studies revealed that the expression of BACH1 was raised in a dose-dependent manner upon ZNF131 overexpression, whose downstream target genes, e.g. SNAI2 or CLDN3 were also up-regulated according to the expression of BACH1 Figure S10C-E).

Desoxyrhaponticin (Deso), an emodin analog, exhibits a range of pharmacological activities both *in vitro* and *in vivo*, including anti-tumor, anti-inflammatory, anti-angiogenic, anti-diabetic, and antibacterial effects, Emodin has exhibited cytotoxic and pro-apoptotic effects against various cancer cell types, including breast, liver, lung, ovarian, prostate, tongue, and pancreatic cancers, primarily by inducing cell cycle arrest (41. Previous studies have shown that emodin reduces the mRNA and protein stability of RAD51 in NSCLC, influencing genes related to apoptosis, tumor metastasis, and chemotherapy resistance, ultimately leading to cell apoptosis 42. These findings align with our experimental results. We further evaluated the safety and efficacy of Deso. The EC50 values were determined for both H1299 cells and normal HBE cells. The EC50 in H1299 cells (86.88μM) was higher than in HBE cells (58.83μM). A concentration of 50μM Deso was used to assess its effect on the proliferation of H1299 cells *in vivo*. The results indicated a significant reduction in tumor volume without affecting the body weight of the mice compared to the control group Figure S11A-B). Based on our findings, 50μM was identified as the minimum effective dose of Deso.

MD assays indicated that Deso may bind to the BTB domain of ZNF131. However, as shown in Figure 7C, deletion of the BTB domain in ZNF131 still conferred significant resistance to both radiation and chemotherapy, indicating a potential alternative mechanism contributing to Deso resistance. To investigate this further, H1299 cells were treated with Deso following the overexpression of ZNF131-FL, ZNF131-∆BTB, or a control vector. Both colony formation and DNA damage assays indicated that the proliferation inhibition and DNA damage induced by Deso were partially rescued by overexpressing ZNF131-FL, but not by ZNF131-∆BTB (Figure S11C-F). Additionally, we treated ZNF131-KO cells with Deso, and the results showed that Deso continued to inhibit proliferation, even in the absence of ZNF131 (Figure S11G-H). These findings suggest that Deso maintains its inhibitory effects at least partially by targeting ZNF131, though additional mechanisms may also play a role in conferring resistance to Deso treatment.

In summary, our investigations unveiled elevated ZNF131 expression in NSCLC, which correlated with advanced TNM stage, lymph node metastasis, and a poor prognosis. ZNF131 overexpression significantly boosted proliferation, invasion, and stemness, both *in vitro* and *in vivo*, by transcriptionally activating RAD51 through its ZF domain. Moreover, the ZNF131-BACH1 interaction via their respective BTB domains appeared to accelerate this process by stabilizing each other and preventing ubiquitin-mediated degradation orchestrated by CUL3, thereby promoting radiotherapy and chemotherapy resistance in NSCLC. Desoxyrhaponticin was shown to halt NSCLC progression and orchestrate a synergistic effect together with chemotherapy partially by inhibiting ZNF131.

## Materials and methods

### Patients and clinical specimens

The study protocol was approved by the Institutional Review Board of the China Medical University. All participants provided written informed consent, and the study was conducted per the principles of the Declaration of Helsinki. This study was subject to approval by the local institutional review board of the China Medical University. Tissue samples were obtained from 109 patients (68 males and 41 females) who underwent complete surgical excision at the First Affiliated Hospital of China Medical University between 2010 and 2012 with a diagnosis of lung squamous cell carcinoma or lung adenocarcinoma, 38 of 109 cases had corresponding non-cancerous lung tissues. No neoadjuvant radiotherapy or chemotherapy was done before surgery. Of the 109 patients, 33 (30.3%) were treated with platinum-based adjuvant chemotherapy, 8 (7.3%) underwent platinum-based adjuvant chemoradiotherapy, and the other 68 patients were treated outside, we did not have information about treatment. The survival of each patient was defined as the time from the day of surgery to the end of follow-up or the day of death. Histological diagnosis and grading were evaluated according to the 2015 World Health Organization (WHO) classification of tumors of the lung (43. All 109 specimens were for histological subtype, differentiation, and tumor stage. Tumor staging was performed according to the seventh edition of the International Union against Cancer (UICC) TNM Staging System for Lung Cancer 44. The median age of 109 patients was 60 years old (ranging from 29 years old to 79 years old). Of the 109 patients, 49 patients were equal to or older than 60 years old, and 60 patients were younger than 60 years old. The samples included 47 squamous cell lung carcinomas and 62 lung adenocarcinomas, respectively. A total of 38 tumors were well differentiated, while 71 were classified as moderately or poorly differentiated. Lymph node metastases were present in 48 of the 109 cases. Our cohort included 83 stage I-II cases and 26 stage III cases. Among 62 adenocarcinoma cases, 24 cases had got KRAS mutation detection, only 1 case harbored KRAS mutant, and 47 had performed EGFR mutation detection, 21 cases harbored EGFR-mutated. Among these cases with EGFR mutation were positive (12 cases in exon 19; 9 cases in exon 21). No ALK mutation was found in all 109 cases. Patient survival was defined as the time from the day of surgery to the end of the follow-up period or the date of death due to recurrence or metastasis. None of the patients had received radiotherapy or chemotherapy before undergoing surgical resection, and all patients were treated with routine chemotherapy after surgery.

An additional 77 (44 male and 33 female) patients, who received neoadjuvant chemotherapy treatment from 2010 to 2021, were included. Of these, all the patients were diagnosed with lung adenocarcinoma. Twenty-three patients were TNM I and 48 patients were TNM II stages, 6 were III stages. Twnenty-nine patients received Pemetrexed + cisplatin plans for 3 cycles 29 received Pemetrexed + Carboplatin plans for 3 cycles, 12 patients received Doxorubicin + Carboplatin plans for 3 cycles, 8 patients received Docetaxel + Carboplatin plans for 3 cycles, 4 patients received Paclitaxel + Carboplatin plans for 3 cycles.

We also selected 100 (80 male and 20 female) patients clinically diagnosed as unresectable primary lung carcinoma and pathologically confirmed as NSCLC who underwent radical intensity-modulated radiotherapy (IMRT) at the Radiation Oncology Department of the First Hospital of China Medical University in Northern China between June 2015 and December 2017. Forty-one patients were diagnosed with Squamous cell carcinoma, and 59 were Adenocarcinoma.20 patients were TNM I and II stages, and 80 were III and IV stages. All patients received IMRT of radical dose (1.8-2.0Gy per fraction, totally 56-60Gy) and repeating CT scan four weeks and 12 weeks after finishing radiotherapy. Target lesions and tumor response were evaluated by Response Evaluation Criteria In Solid Tumors (RECIST) 1.1. All patients underwent CT scans four weeks after finishing chemotherapy. Target lesions and tumor response were evaluated by Response Evaluation Criteria In Solid Tumors (RECIST) 1.1^1^. Complete response (CR) and partial response (PR) were regarded as good efficacy, and stable disease (SD) and progressive disease (PD) were defined as poor outcomes in this study 45.

A total of 16 freshly isolated specimens, including both tumor tissue and the corresponding normal tissues, were stored at -70°C immediately after resection for subsequent protein extraction.

### Functional enrichment analyses

This was performed as described by Li *et al.* 45. DAVID (https://david.ncifcrf.gov/summary.jsp), an online tool for gene functional enrichment, was used for GO analysis (concerning cellular components, molecular function, and biological process) and the KEGG pathway analysis of the DEGs shared between high and low expression of ZNF131 group. The results were displayed using the ggplot2 R package. P < 0.05 was considered statistically significant.

GSEA was adopted to identify the signaling pathway of elevated ZNF131-related gene signature in NSCLC patients. P < 0.05 was considered statistically significant. The pathways used for GSEA were obtained from the Molecular Signatures Database (http://software.broadinstitute.org/gsea/_msigdb).

### Cell culture

The HBE cell line was obtained from the American Type Culture Collection (ATCC; Manassas, VA, USA). NSCLC cell lines A549, H1299, H460, H358, SK-MES-1, and NCI-H1975 were obtained from Shanghai Cell Bank (Shanghai, China). The LK2 cell line was obtained from a gift from Dr. Hiroshi Kijima (Department of Pathology and Bioscience, Hirosaki University Graduate School of Medicine, Japan). Cells were stored as frozen aliquots. All cell lines were authenticated using short tandem repeat DNA profiling with no more than 10 passages and no mycoplasma contamination. For experiments, all cells were cultured in RPMI 1640 (Invitrogen, Carlsbad, CA, USA) containing 10 % fetal calf serum (Invitrogen), 100 IU/mL penicillin (Sigma, St. Louis, MO, USA), and 100 μg/mL streptomycin (Sigma) in sterile culture dishes. Cultures were passaged every 2 days using 0.25 % trypsin (Invitrogen); experiments were conducted using cells within 10 passages.

### Immunohistochemistry

Immunohistochemistry analysis was conducted as described earlier 46. Tissue sections were incubated with rabbit monoclonal antibodies against ZNF131 (1:50, Sigma, St. Louis, MO, USA), RAD51 (1:100; Proteintech, Chicago, IL, USA), and Ki-67 (1: 50; Cell Signaling Technology, Danvers, MA, USA). Each section was evaluated and scored independently by 2 pathologists. A semiquantitative scoring system was used in this assay. Intensity was scored as: 0, negative; 1, weak; 2, moderate; and 3, strong. The percentage of stained cells was scored as: 1, 1-25%; 2, 26-50%; 3, 51-75%; and 4, 76-100%. The scores of each tumor sample were multiplied to give a final score from 0 to 12; The overall score < 4 was defined as negative and ≥ 4 as positive. Detailed descriptions are mentioned in the Supplementary materials and methods.

### Western blotting, proteasome-inhibition and ubiquitination assays and immunoprecipitation

Western blotting and immunoprecipitation were performed as described in the study by Hu *et al.* 47. Total protein was extracted using a lysis buffer (Pierce, Rockford, IL, USA) and quantified with the Bradford method 48. Fifty μg of the total protein samples were separated by 10% SDS-PAGE and transferred onto polyvinylidene fluoride membranes (PVDF; Millipore, Billerica, MA, USA). Membranes were incubated overnight at 4°C with the following primary antibodies: GAPDH and ZNF131 (mouse polyclonal, # H00007690-B0IP, Abnova), Myc-tag, DYKDDDDK Tag, HA-tag, GST-tag, RAD51, γ-H2AX (1:1000; Cell Signaling Technology, Danvers, MA, USA), BACH1 (1:1000, 14018-1-AP, Proteintech, Chicago, IL, USA), BACH1 (1:1000, SC-271211) and CUL-3 (sc-166110, Santa Cruz, CA, USA), GFP antibody (1:1000, Abcam, Cambridge, UK ab290). Membranes were washed and subsequently incubated with peroxidase- conjugated anti-mouse or anti-rabbit IgG (Santa Cruz Biotechnology) at 37 °C for 2h. Bound proteins were visualized using electrochemiluminescence (Pierce, Rockford, IL, USA) and detected with a bio-imaging system (DNR Bio-Imaging Systems, Jerusalem, Israel). Detailed descriptions are mentioned in the Supplementary materials and methods.

### Reagents

pCMV6-ZNF131-Myc, pCMV6-ZNF131-ΔBTB-Myc, pCMV6-ZNF131-ΔDisorded-Myc, pCMV6-ZNF131-ΔZF-Myc, pCMV6-ZNF131-K18R-K46R-K49R-Myc, pCMV6-ZNF131-K57R-K61R-K79R-Myc, pCMV6-ZNF131-K107R-K119R-K126R-Myc, pCMV6-ZNF131-K49R-Myc, pCMV6-ZNF131-K126R-Myc, pLentiCRISPRV2-U6-ZNF131-sgRNA-cas9 #1 (GATATCTTCGAGAGAGCGCA-TG), #2 (AATCGTGCACCTGGCTGTCCTG), and control sgRNA (GCACTACCAGAGCTAAC-TTCA), pLV2-CMV-BACH1-3xFLAG, pLV2-CMV-BACH1-ΔBTB -3xFLAG, pLV2-CMV-BACH1-ΔDisorded-3xFLAG, pLV2-CMV-BACH1-ΔbZIP-3xFLAG, PCMV-EGFP-CUL3-Neo, pLenti-CRISPRV2U6-RAD51-sgRNA-cas9,pLV2-CMV-BACH1-K29R-K30R-3xFLAG,pLV2-CMV-BACH1-K95R-K100R-3xFLAG, pLV2-CMV-BACH1-K108R-3xFLAG were purchased from MiaoLingBio (Wuhan, China). Lipo3000 (Invitrogen, Carlsbad, CA, USA) was used for transfection. MG132 (HY-13259), cycloheximide (20ug/ml, HY-12320), Cisplatin (HY-17394), and RAD51 Inhibitor B02 (HY-101462) were purchased from MedChemExpress (Monmouth Junction, NJ, USA).

### Immunofluorescence and EdU assay

The assay was conducted as described by Hu *et al.* 47. HBE, A549, NCI-H1975, H460, LK2, H1299, SK-MES-1 and H358 cells were incubated with antibodies against ZNF131 (1:50, Abnova). BACH1 (14018-1-AP) was purchased from Proteintech (Chicago, IL, USA). BACH1 (SC-271211) and were from Santa Cruz Biotechnology (Santa Cruz, CA, USA). γ-H2AX (1:50, Cell Signaling Technology, Danvers, MA, USA). The average number of γ-H_2_AX foci per nucleus was examined at least 100 nuclei. The fluorescent image is imported into Fiji software and the ROI tool is used to circle the range to be measured to remove the background influence. Then use the co-location threshold of the co-location analysis plug-in built-in Fiji software (http://fiji.sc) for co-location analysis 34. Detailed descriptions are provided in Supplementary materials and methods.

### MTT, colony formation assay

The MTT and colony formation assays were performed as described by Zhang *et al.* 46. Detailed descriptions are provided in Supplementary materials and methods.

### Flow cytometry

Cells (5 × 10^6^) were seeded into 6-cm tissue culture dishes. Twelve hours later, cells were transfected with ZNF131 plasmid or empty vector and ZNF131-sgRNA or NC-sgRNA. Forty-eight hours after transfection, cells were harvested, fixed in 1% paraformaldehyde, washed with phosphate-buffered saline (PBS), and stained with 5 mg/ml propidium iodide in PBS supplemented with RNase A (Roche, Indianapolis, IN) for 30 min at room temperature. Data were collected using BD systems. One-parameter histogram was plotted according to the distribution of nuclear DNA content in each cell detected by a flow cytometer. Cells in each phase of the cell cycle were determined based on their DNA ploidy profile.

### Sphere formation assay

For sphere formation assay, 1 × 10^3^ cells were cultured in a 24-well low-attachment surface polystyrene culture plate (Costar, Cambridge, MA, USA) using serum-free DMEM-F12 (Invitrogen, Carlsbad, CA, USA), containing 1× B27 (Invitrogen, Carlsbad, CA, USA), 20 ng/mL EGF (BD Bioscience, San Jose, CA, USA), and 4 mg/mL insulin (Sigma, St. Louis, MO, USA) at 37 °C and 5% CO_2_ for 10-14 days. Mammospheres with a diameter > 75 µm in five randomly selected fields were counted.

### Transwell assay

Assays were performed as described by Zhang *et al.* 46. Detailed descriptions are provided in Supplementary materials and methods.

### RNA extraction and real-time RT-PCR

The protocols are described in a previous publication 45. Primer sequences are listed in Table S3.

### Luciferase reporter assay

The assay was performed as previously described 45. Detailed descriptions are provided in Supplementary materials and methods.

### RNA-sequencing array

Total RNA was extracted from H1299 and H1299-CDDP-resistant cells or control and ZNF131-overexpressing cells using TRIzol reagent (Takara, Kyoto, Japan) according to the manufacturer's instructions. RNA purity was determined using a NanoPhotometer spectrophotometer (IMPLEN, Westlake Village, USA). cDNA libraries were constructed from 1 μg of total RNA using a PCR-cDNA Sequencing Kit (SQK-PCS109; Nanopore Technologies, Oxford, UK) according to the manufacturer's protocol. Genes with fold change ≥ 2.0, identified using DESeq, were designated as “differentially expressed.” All operations were performed using Biomarker Technologies (www.bionarker.com.cn).

### RNA-microarray

One qPCR array was designed to analyze 15 TFs differentially expressed between H1299 and H1299-cisplatin-resisting cells, another qPCR array was designed to analyze the 85 DEGs after the ChIP-sequencing array, RNA-sequencing array, and GSEA, according to manufacturer instructions (Wcgene Biotechnology Corporation, Shanghai, China). Genes that could not be detected three times were excluded. The Ct values for each gene were corrected using Ct readings of GAPDH.

### Chromatin immunoprecipitation (ChIP)

H1299 with overexpression of ZNF131 was inoculated on a 15cm petri dish, and 24 h later 1% formaldehyde cross-linked for 10 min, then operated according to the instructions of the Chip kit (Millipore). In short, a mixture of cellular formaldehyde is ultrasound-treated on ice to obtain DNA fragments with an average length of 200-800bp. One percent of each sample was saved as an input fraction. Then immunoprecipitation was performed using Myc-tag antibody (Cell Signaling Technology, #2276) or IgG was used as control. The DNA for RAD51 or MCM2 obtained after reverse crosslinking was dissolved in 50ul of Nuclease-Free water (Invitrogen) and qPCR analysis was performed using the specific primers in Table 5 or Table 6. The normalization method for ChIP analysis is the percent of input. Each ChIP result represents the average of 4 samples (mean ± SD). For P-value computation, the student t-test was applied. After that, the samples obtained by PCR were subjected to Agarose Gel Electrophoresis.

### ChIP-sequencing array

ChIP-Seq was carried out by conventional ChIP followed by end repair of 15 ng (for protein-GFP fusions) or 30 ng (for the histone modifications). Adaptors were ligated to DNA fragments, which were subsequently size selected (∼300 base pair (bp). The adaptor-modified DNA fragments were subjected to limited PCR amplification (14 cycles) and quality control was made by qPCR (primers sequences are available upon request), as well as by running the PCR products on a Bioanalyzer (Bio-Rad). Finally, cluster generation and sequencing-by-synthesis (36 bp) were performed using the Illumina Genome Analyzer IIx (GAIIx) according to standard protocols of the manufacturer (Illumina). The image files generated by the Genome Analyzer were processed to extract DNA sequence data. Sequences were aligned to the human reference genome (GRCh37/hg19, Feb 2009) with Burrows-Wheeler Aligner1 (bwa, v0.5.9-r16) allowing one mismatch. Uniquely aligned reads were converted to BED format. The total number of sequenced fragments and mapped fragments are shown in supplemental Table S4. All ChIP and input samples were normalized randomly to the same number of reads 49. Furthermore, reads were directionally extended to 300 bp, and for each base pair in the genome, the number of overlapping sequence reads was determined and averaged over a 10-bp window to create a Wiggle (WIG) file to visualize the data in the University of California Santa Cruz (UCSC) Genome Browser.

### LC-MS/MS analysis

ZNF131 overexpression proteins in the gel pieces from co-immunoprecipitation (co-IP) were analyzed by nano-LC-MS/MS on a Q Exactive mass spectrometer (Thermo Fisher Scientific) coupled with an Easy nLC system (Invitrogen, Carlsbad, CA, USA). Raw MS/MS data were converted into MGF format using Proteome Discoverer 1.4 (Invitrogen, Carlsbad, CA, USA). Peptide identification was performed using Mascot software (Version 2.3.01, Matrix Science, UK) with the UniProt database search algorithm and an integrated FDR analysis function. The data were used to conduct searches against a protein sequence database downloaded from the 2021_uni_mus (128,510 sequences; 62,817,431 residues). The MS/MS spectra were searched against a decoy database to estimate the false discovery rate (FDR ˂ 0.05) for peptide identification.

### GST pull-down assay

The ZNF131 protein coupled to a GST label was induced in Escherichia coli BL21 (30° C, 3 h, 200 rpm/min) and purified according to standard steps. The purified protein was recombined with glutathione sepharose (GE Healthcare, Waukesha, WI, USA) magnetic beads and then incubated overnight with H1299 cell lysate transfected with BACH1 plasmid at 4°C. Finally, the complexes were detected by western blotting and Coomassie brilliant blue staining.

### Linear-quadratic model

The assay was performed as previously described 34. Cell survival was quantified using the colony formation assay for a dose range from 0 to 8 Gy for the combination of radiation with or without ZNF131. Cells were seeded into 6-well plates with cell densities depending on the dose of radiation received; 0-2Gy: 500 cells, 4Gy: 1,000 cells, and 8Gy: 2,000 cells. The plating efficiency (PE) was calculated to be the percentage of the cells plated to develop colonies. The value of PE was subsequently used to determine the surviving fraction (SF) for each dose, as the proportion of colonies counted compared with controls. Graphs of radiation dose against surviving fraction were then fitted with the Linear-Quadratic Model (α and β values are the linear and quadratic components of cell killing). Each experiment was repeated in triplicate.

### Transplantation of tumor cells into nude mice

The animals were treated according to the National Institutes of Health Guidelines for the Care and Use of Laboratory Animals (NIH Publication No. 8023, revised 1978). Nude mice were treated according to the experimental animal ethics guidelines issued by the China Medical University (CMU2021731). The assay was performed as previously described 45. Each mouse was randomly assigned to each group and the investigator was blinded to the group allocation. A mouse model of xenografts was established using adult female nude mice, which were injected with 2 × 10^5^ H1299 cells subcutaneously (“primary” or “irradiated” tumor) on day 1. Tumor-bearing mice were sorted into groups according to the mean primary tumor volume before treatment with radiation, which was delivered to primary tumors in three fractions of 8 Gy (8 Gy × 3) on days 10, 11, and 12 after inoculation. For locally irradiated primary tumors, mice were placed on a jig, which shielded their entire body except for the leg bearing the targeted tumor, thus allowing a negligible abscopal radiation effect.

For chemotherapy treatment, after one-week tumor inoculation, the mice were randomly divided into DMSO + Vector, DMSO + ZNF131, CDDP + Vector, CDDP + ZNF131, DMSO + DMSO, CDDP + DMSO, DMSO + Desoxyrhaponticin, and CDDP + Desoxyrhaponticin groups, respectively. CDDP and Desoxyrhaponticin were administered every 3 days at a concentration of 5 mg/kg 50 and 40 mg/kg 51, 52 respectively.

For the limiting dilution injection, 8-week-old nude female recipient mice (for H1299 cell injection) were anesthetized with isoflurane. H1299 NSCLC cells were suspended at a different density into a 1:1 mixture of DMEM and Matrigel (BD Biosciences). Cells were injected into nude mice at 3 × 10^4^, 1 × 10^4^, 3 × 10^3^, or 1 × 10^3^ cells. Tumor volume was calculated according to the formula: volume = length × width × width × 0.5. The tumors were fixed in 4% formaldehyde (Sigma), and embedded in paraffin.

For investigating the minimum concentration of Deso, after one-week tumor inoculation, the mice were randomly divided into 5 groups, Desoxyrhaponticin was administered every 3 days at diverse concentrations of 25μM, 50μM,100μM and 200μM respectively.

### Preparation of the protein and ligand

The 3D structure of ZNF131 was predicted by inputting its protein sequence on AlphaFold2. Then, to optimize the crystal structure of the obtained proteins, the Protein Preparation Wizard module of Schrödinger software was used to perform protein preprocess, regenerate states of native ligands, H-bond assignment optimization, protein energy minimization, and, respectively remove waters.

The 2D SDF structure files for 35,100 compounds were downloaded and processed from 8 compound libraries, which were then processed using the LigPrep module in Schrödinger, and all 3D chiral conformations were generated. Finally, drug-molecules were geometry minimized using OPLS3e force-field 53.

### Molecular docking

The SiteMap module in Schrödinger was used to predict the optimal binding site, and then the Receptor Grid Generation module was used to set the most appropriate Enclosing box to perfectly wrap the predicted binding site, and the active pocket of the protein was obtained on this basis. Each of the processed ligands in the 8 compound libraries was molecularly docked with the active sites of ZNF131 protein (HTVS, SP, and XP were used respectively, and the docking accuracy was gradually improved), and the ligands with the lowest score were calculated and analyzed by MM-GBSA with the active sites of ZNF131 protein, and the ligands that were stably bound to ZNF131 protein were found according to the level of binding free energy.

### Molecular dynamics simulation

To further optimize the binding mode of compound-protein complexes, we perform conventional molecular dynamics simulations using the Desmond program. The OPLS4 forcefield was employed to parameterize the protein and small molecules, while the SPCE model was used for the water solvent. The protein-small molecule complex was placed in a cubic water box and solvated. The system's charge was neutralized by adding 0.150 M chloride and sodium The energy of the system was initially minimized using the steepest descent minimization method for 50,000 steps. Subsequently, the positions of heavy atoms were restrained for NVT and NPT equilibration for an additional 50,000 steps. The system temperature was maintained at 300 Kand the system pressure was maintained at 1 bar. After completing the two equilibration stages, an unrestricted simulation was performed for 100 ns. The interactions were analyzed and dynamic trajectory animations were generated using Maestro 2023.

### Statistical analyses

All data were analyzed using SPSS version 22.0 (Chicago, IL, USA). The chi-square test was used to evaluate the correlation between ZNF131 expression and clinicopathological factors. Kaplan-Meier survival curves were plotted, and the log-rank test was performed. Spearman's correlation analysis was performed to examine the correlation between ZNF131 and RAD51 expression. All clinicopathological parameters were included in the Cox regression model and assessed by univariate analysis using the enter method. The student's t-test was used to analyze differences between the groups. One-way analysis of variance (ANOVA) was used to compare multiple groups. All experiments were performed in triplicate. A P-value less than 0.05 was considered statistically significant.

## Supplementary Material

Supplementary figures and tables.

## Figures and Tables

**Table 1:** Correlation of the cytosolic overexpression of ZNF131 with clinicopathological features in 109 cases of NSCLC

Clinicopathological	N	Positive	Negative	χ2	*P*
Factors					
Age (years)					
< 60	60	34	26	0.495	0.251
≥ 60	49	22	27		
Gender					
Male	70	38	32	0.663	0.432
Female	39	18	21		
Histological type					
Squamous cell carcinoma	49	28	21	1.185	0.337
Adenocarcinoma	60	28	32		
Differentiation					
Well	53	23	30	2.630	0.127
Moderate + Poor	56	33	23		
Tumor size				3.704	0.077
≥ 3cm	66	29	37		
< 3cm	43	27	16		
TNM classification					
Ⅰ+Ⅱ	71	31	40	4.851	0.044
Ⅲ	38	25	13		
Lymph node metastasis					
Positive	45	17	28	5.673	0.02
Negative	64	39	25		

**Table 2:** Summary of Cox univariate and multivariate regression analysis of the association between clinicopathological features and overall survival in 109 cases of non-small cell lung cancer (NSCLC)

Clinicopathological	Hazard ratio	*P*
Feature	(95% CI)	
**Univariate analysis**		
Age	0.885(0.485-1.866)	0.952
Gender	1.189(0.604-2.338)	0.617
Histological type	0.842(0.434-1.634)	0.610
Differentiation	1.694(0.860-3.337)	0.128
Tumor size	3.829(1.910-7.675)	< 0.001
TNM classification	4.099(2.067-8.131)	< 0.001
Lymph node metastasis	10.738(3.280-35.159)	< 0.001
ZNF131 expression	2.706(1.296-5.650)	0.008
**Multivariate analysis**		
Tumor size	2.530(1.245-5.142)	0.010
TNM classification	1.695(0.802-3.583)	0.167
Lymph node metastasis	5.877(1.638-21.085)	0.0007
ZNF131 expression	1.609(0.748-3.462)	0.223

**Table 3:** Parameters of radiobiology fitted linear quadratic model

	α	95% CI(α)	β	95% CI(β)	α/β
NC	0.2666	0.2066 to 0.3270	0.01160	-0.001103 to 0.02628	22.99
ZNF131-FL	0.1661	-0.01175 to 0.3490	0.003433	-0.02616 to 0.04417	48.40
ZNF131-ΔBTB	0.1638	0.1119 to 0.2163	0.01757	0.007063 to 0.02925	9.320
ZNF131-ΔZF	0.2537	0.2015 to 0.3059	0.01463	0.003234 to 0.02762	17.35

**Table 4:** Correlation of ZNF131 with the expression of RAD51 in 100 NSCLC specimens

			ZNF131			*r*		*P*
		Negative		Positive				
RAD51	Negative	37		15	0.525		< 0.001	
	Positive	9		39				
								

**Table 5:** Primers of RAD51 used for ChIP assay

Primers	For Primer5'-3'	Rev Primer5'-3'
1	ggagggaaaatagatctaacc	tacaggcatgcaccaccacgc
2	tcaagcacttctctgcctcag	tgtcttttgagacaaggtctc
3	gccaccgcgcctggcctctcc	gctggtctccaactcctgagc
4	ctgagaccacaggcacaagcc	gccagataatactaatcttta
5	ccttccagtttcggcacttgc	caagcgattctcatgcctcag
6	cggcgagatctcggttggctg	ctcagcctttaaaaccggaat
7	ctcctaaactgctgggattac	cagctcctccacctccatgag
8	acgctagctccatttcccact	cctcttgggagtcgtggtctt
9	ccgggagatgtagtcccgggc	cataaagtttgaattagtcct
10	cagctgggactacacgcgtga	cgtcgacgcgggcgtgaccct
11	cagctgggactacacgcgtga	ctgggcgagagggtttggcggg

**Table 6:** Primers of MCM2 used for ChIP assay

Primers	For Primer5'-3'	Rev Primer5'-3'
1	gcatgcgccactacgcctggc	gtctcttaactgttggggtttc
2	tcctgtcagcagctcctagca	ttaaattgctggtcttcctgt
3	gccagaaacaaactcatcttc	atggtgaaatcccatctctact
4	ccacttgtcaggagttggaga	tctctccatcactcctgcctcc
5	cactccagcctgggtgacaga	cacttccacctggtagcagcca
6	ctggaactagacaagaaccac	gaggggcttgcacatccgggc
7	tgcccggcatcataagctccta	ccaggctctgaacctctgctgt
8	cattgtgctcctaagacggtaa	tcgaactcctgagcttgtgatc
9	gtttcaccatgttagtcaggc	tccagctcccaccacagcgca
