# Supplementary material for: ZNF131-BACH1 transcriptionally accelerates RAD51-dependent homologous recombination repair and therapy-resistance of non-small-lung cancer cells by preventing their degradation from CUL3
Source: Theranostics. 2024 Oct 28;14(18):7241–64. doi: 10.7150/thno.97593 (PMC11610138; doi:10.7150/thno.97593)
Supplement: Supplementary file 1 — Supplementary figures and tables. [file thnov14p7241s1.pdf]

Supplementary Figure S1-S11

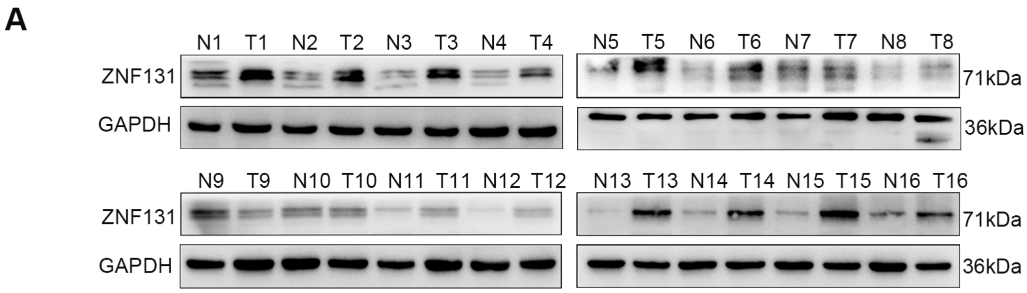

Figure S1. ZNF131 Is Highly Expressed in NSCLC Samples

(A) Western blotting assay was performed to assess the protein levels of ZNF131 in 16 paired fresh non-small cell lung cancer (NSCLC) samples.

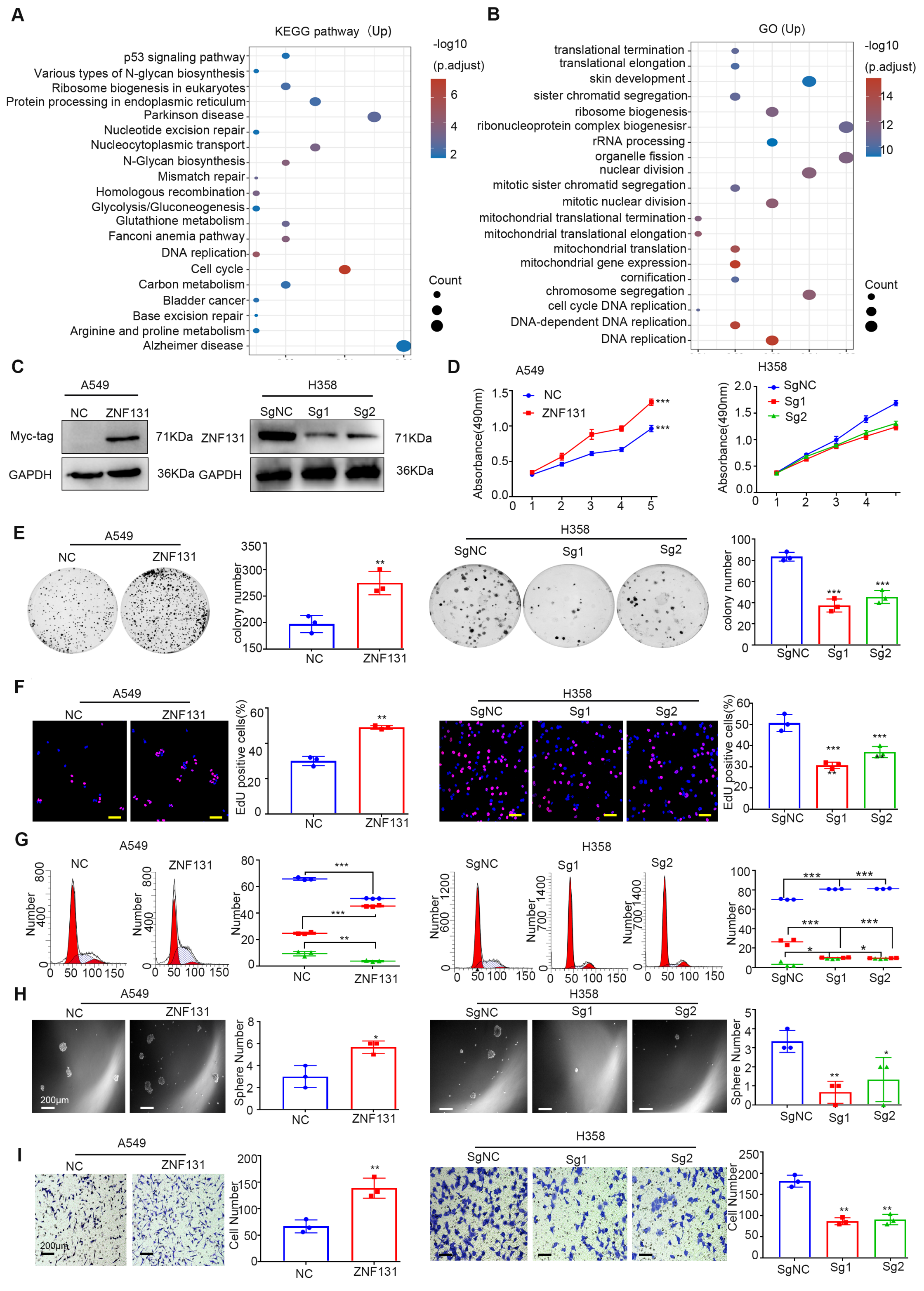

Figure S2. Overexpression of ZNF131 Promotes NSCLC Proliferation and Invasion

(A-B) Gene Set Enrichment Analysis (GSEA) and KEGG pathway analysis of ZNF131 in non-small cell lung cancer (NSCLC) from the TCGA database. (C) Western blot analysis to assess the

overexpression efficiency of ZNF131 in A549 cells and knockout efficiency of TMEM120B in H358 cells. (D-I) The effects of ZNF131 overexpression or silencing on NSCLC cell proliferation, stemness, and invasion were evaluated using the following assays: MTT assay (D), colony formation assay (E), EDU assay (F, scale bar = 100 μm), flow cytometry (G), sphere formation assay (H), and transwell assay (I). Quantitative data are presented as Mean ± SD from three independent experiments. Statistical significance was determined using two-sided t-tests (* $P < 0.05$, ** $P < 0.01$, *** $P < 0.001$).

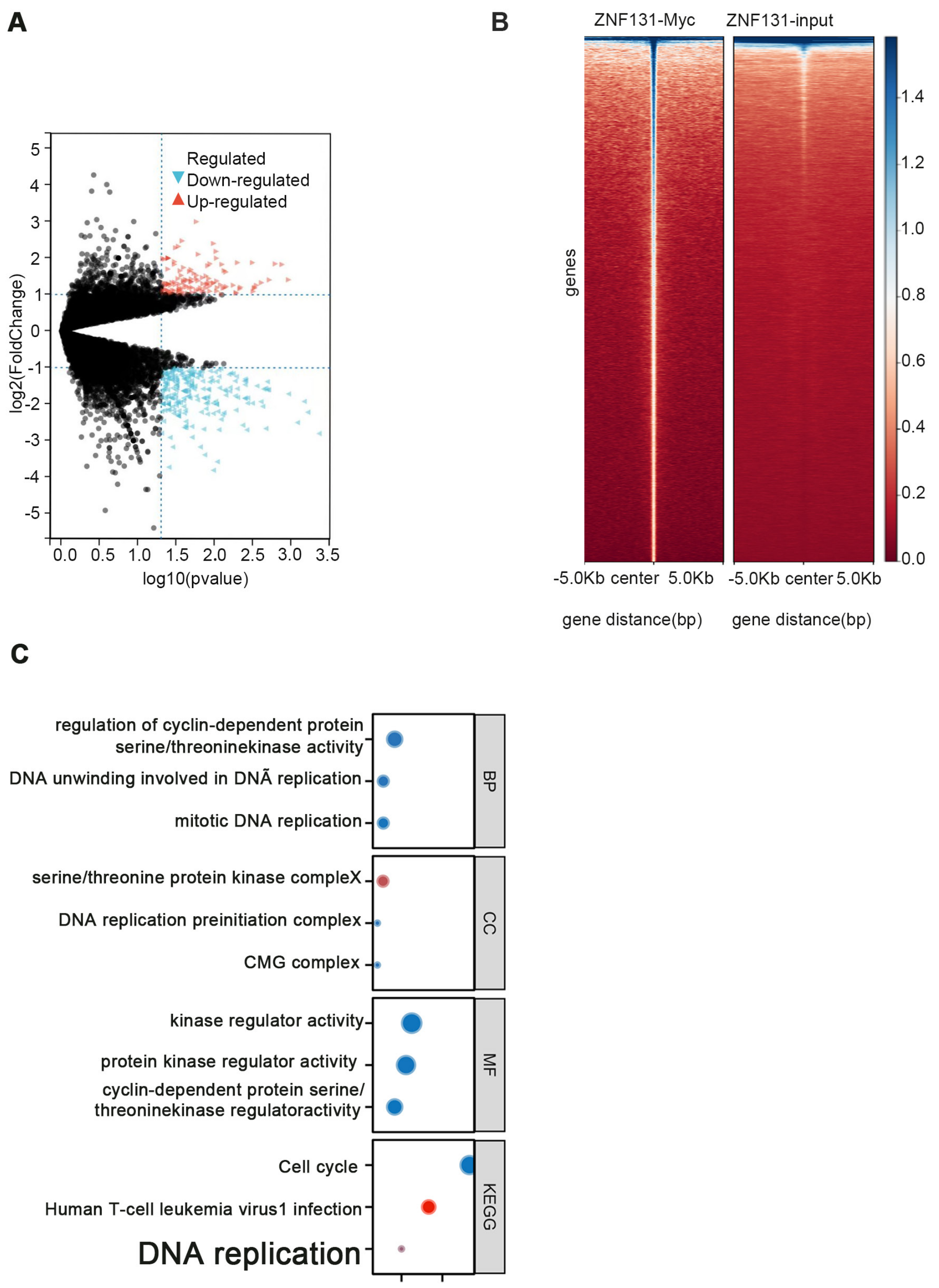

Figure S3. ZNF131 Transcriptionally Upregulates RAD51

(A) Volcano plot showing differentially expressed genes identified by RNA sequencing after overexpression of ZNF131. (B) Heatmap displaying differentially expressed genes identified by ChIP-sequencing after ZNF131 overexpression. (C) Gene Ontology (GO) analysis of the 85 differentially expressed genes identified through the integration of ChIP-sequencing, RNA sequencing, and GSEA.

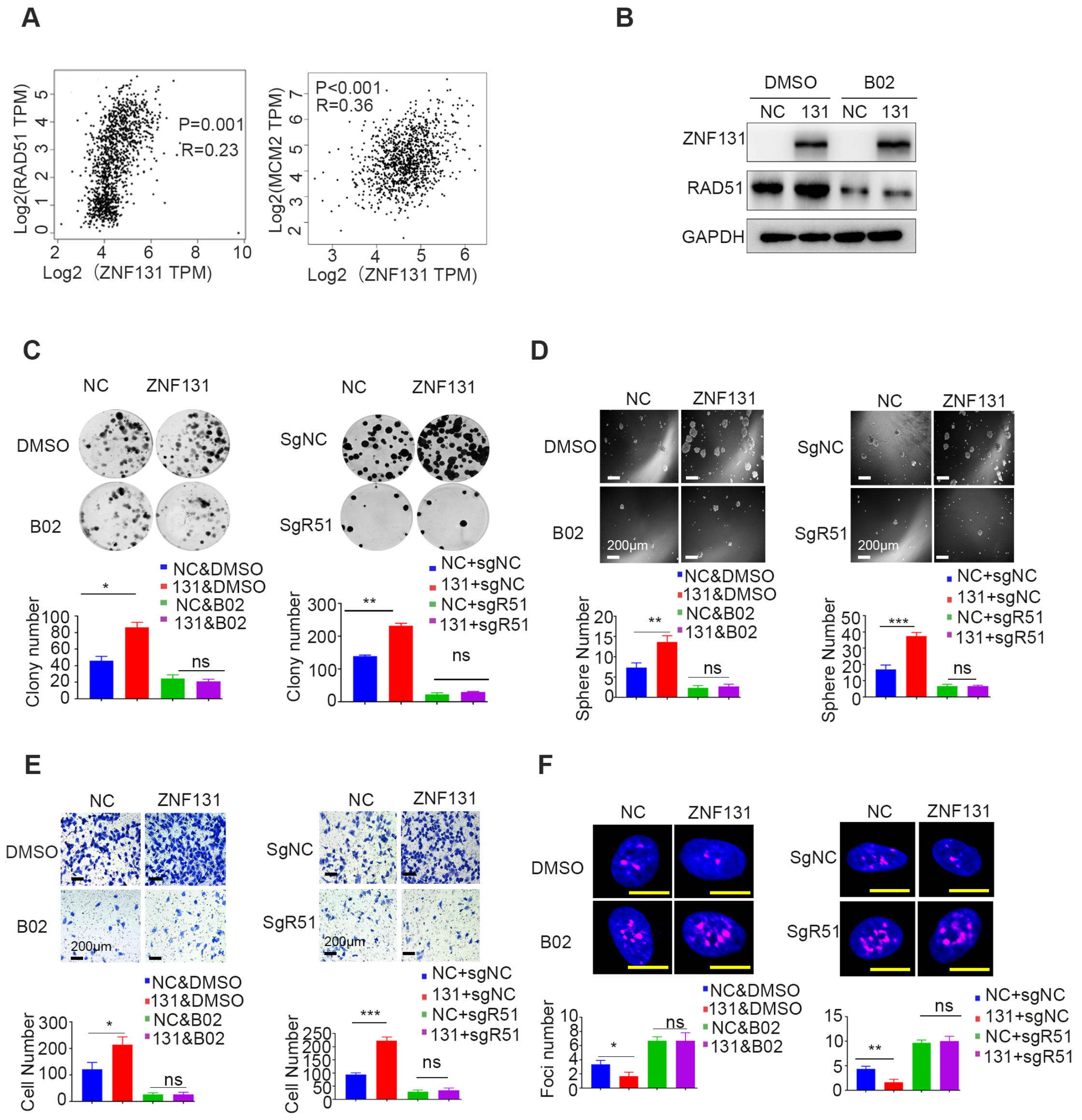

Figure S4. ZNF131 Promotes Lung Cancer Proliferation, Invasion, and Homologous Recombination by Increasing RAD51

(A) Analysis of the correlation between ZNF131 and RAD51 or MCM2 using the GEPIA database. (B) Western blot analysis to detect protein levels of ZNF131 and RAD51. (C-F) The effects of ZNF131 overexpression, combined with RAD51 deletion using sgRNA or the addition of the RAD51-specific inhibitor B02, on proliferation, invasion, and homologous recombination were evaluated using the following assays: colony formation (C), sphere formation (D), transwell (E), and immunofluorescence (F, scale bar = 100 μm). Quantification data are presented as Mean ± SD from three independent

experiments. Statistical significance was determined using two-sided t-tests (* $P < 0.05$, ** $P < 0.01$, *** $P < 0.001$).

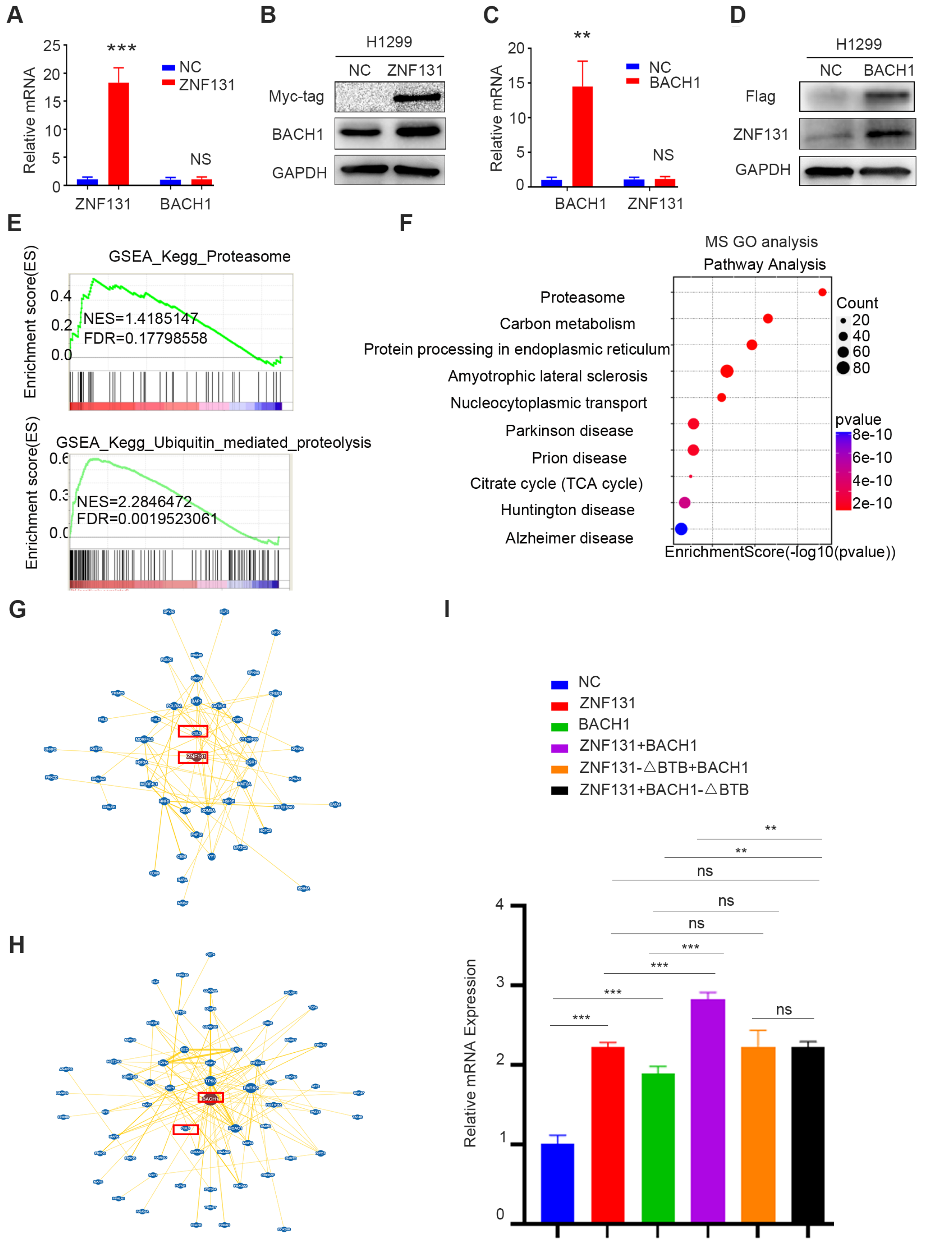

Figure S5. ZNF131 and BACH1 Stabilize Each Other by Preventing Degradation via CUL3

(A-D) Western blot and qPCR assays were performed to assess the protein and mRNA levels of ZNF131 and BACH1 upon overexpression or silencing of ZNF131 and BACH1 (sgRNA), respectively.

(E) GSEA indicated that differential genes associated with high ZNF131 expression were enriched in

the proteasome and ubiquitin-mediated proteolysis pathways. (F) GO analysis for potential candidates identified through mass spectrometry (MS) analysis. (G-H) Protein interaction network for both ZNF131 and BACH1. (I) qPCR assays were performed to assess the mRNA levels of RAD51 after transfection with ZNF131-myc, ZNF131-ΔBTB-myc, BACH1-flag, BACH1-ΔBTB-flag, alone or in combinations: ZNF131-myc + BACH1-flag, ZNF131-ΔBTB-myc + BACH1-flag, or ZNF131-myc + BACH1-ΔBTB-flag. Quantification data are presented as mean ± SD from three independent experiments. Statistical significance was determined using two-sided t-tests (** $P < 0.01$, *** $P < 0.001$).

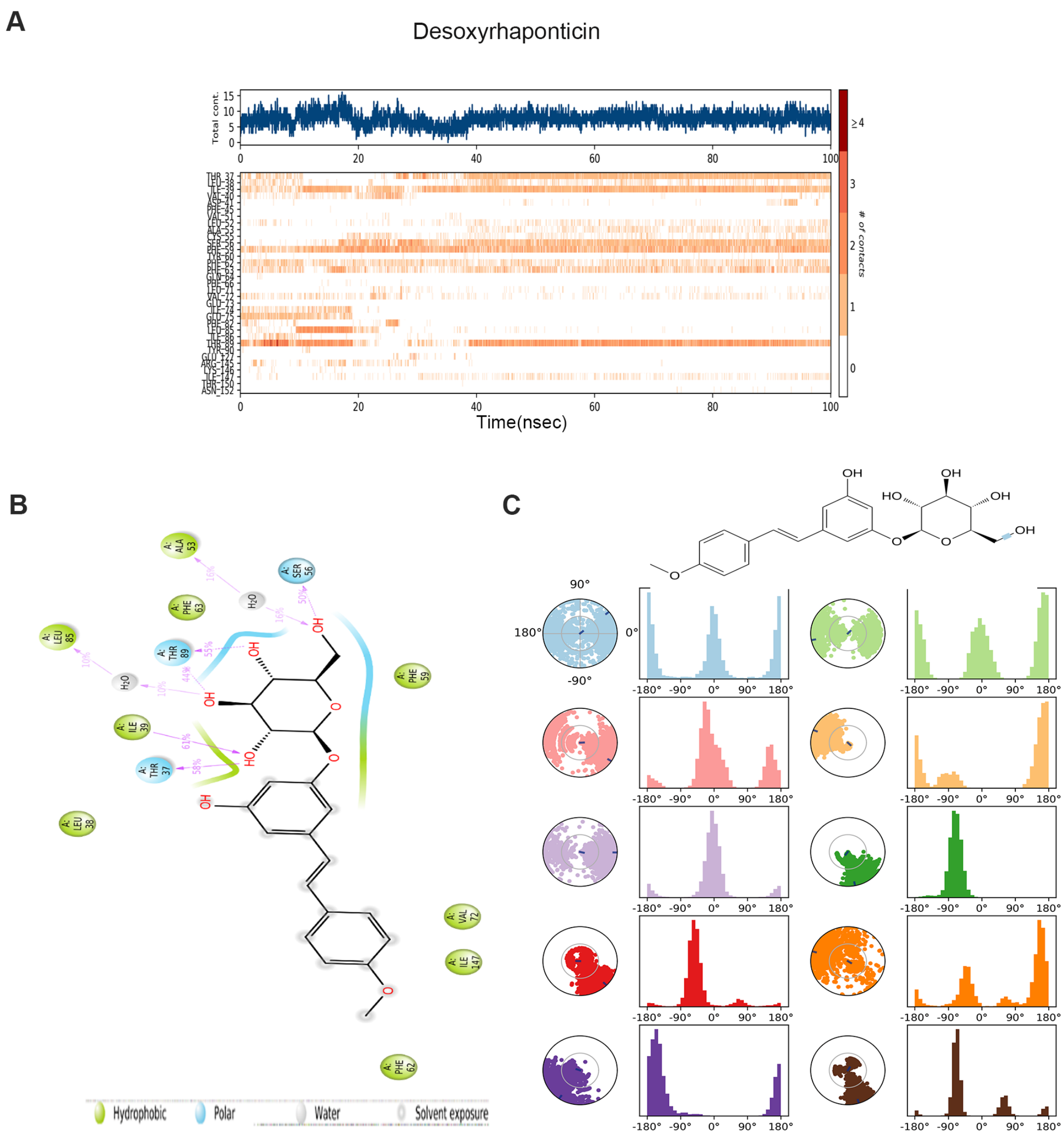

Figure S6. Pharmacokinetics Simulation of Desoxyrhaponticin

(A) The amino acid residues THR37, ILE39, SER56, PHE59, PHE62, PHE63, and THR89 of the ZNF131 protein form close binding interactions with Desoxyrhaponticin. (B) A detailed schematic diagram illustrating the interactions between Desoxyrhaponticin and the ZNF131 protein residues. (C) The ligand torsional diagram shows the conformational evolution of each rotatable bond (RB) of Desoxyrhaponticin throughout the entire simulation trajectory. The dial (or radial) diagram describes the torsional conformations during the simulation process, and the accompanying bar chart summarizes the data from the dial diagram, displaying the probability density of the torsional states.

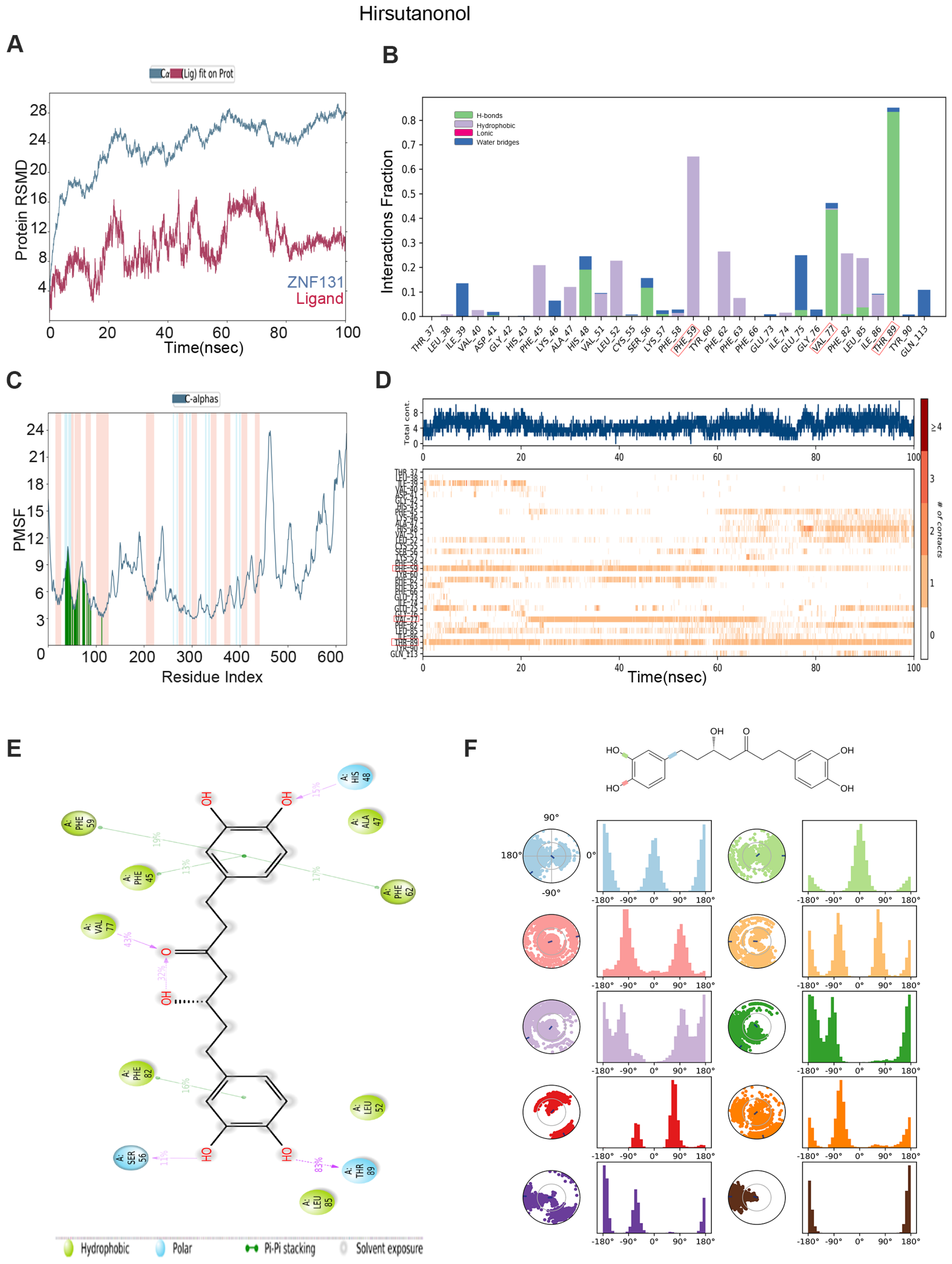

Figure S7. Pharmacokinetics Simulation of Hirsutanonol

(A) The MD simulation of Hirsutanonol binding to ZNF131 protein was performed over 100 ns, and the molecular dynamics trajectories were analyzed. The results showed that the Hirsutanonol-ZNF131 complex became relatively stable after 80 ns, indicating the system reached equilibrium. (B) The

RMSF (Root Mean Square Fluctuation) analysis revealed that Hirsutanonol binding induced high structural flexibility in the 450-470AA and 550-600AA regions of the ZNF131 protein. (C) Interaction analysis identified that PHE59, VAL77, and THR89 are the key amino acids involved in the binding of Hirsutanonol to ZNF131. The interactions mainly involve hydrophobic forces, hydrogen bonding, and water bridges. (D) The amino acid residues PHE59, PHE62, VAL77, and THR89 of ZNF131 protein are tightly bound to Hirsutanonol. (E) A detailed schematic representation of the interactions between Hirsutanonol and the residues of ZNF131. (F) The ligand twist plot illustrates the conformational evolution of each rotatable bond (RB) of Hirsutanonol during the entire simulation trajectory. The dial (or radial) plot depicts the torsional conformations throughout the simulation, while the accompanying bar graph summarizes the data, showing the probability density of the conformational twists.

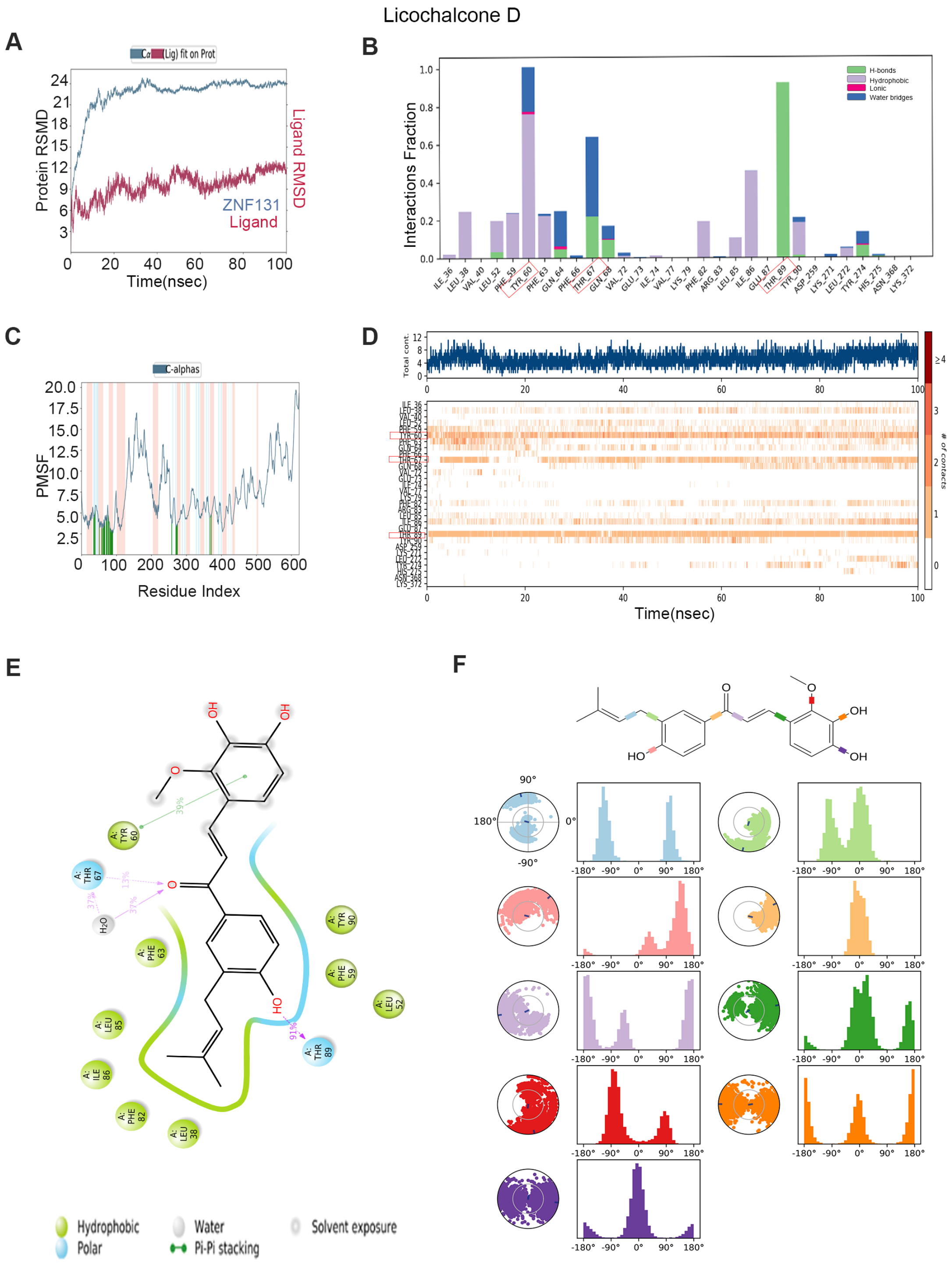

Figure S8. Pharmacokinetics Simulation of Licochalcone D

(A) MD simulation of Licochalcone D bound to ZNF131 over 100 ns. Analysis of the molecular dynamics trajectories shows that the Licochalcone D-ZNF131 complex became relatively stable after 20 ns, indicating that the system reached equilibrium. (B) RMSF (Root Mean Square Fluctuation)

results showed that Licochalcone D binding caused high structural flexibility in the ZNF131 protein, particularly in the 130-190AA and 520-600AA residue regions. (C) Interaction analysis identified THR60, THR67, and THR89 as key amino acids involved in the binding of Licochalcone D to ZNF131. The interactions mainly involve hydrophobic forces, hydrogen bonds, and water bridges. (D) The amino acid residues THR60, THR67, ILE86, and THR89 of ZNF131 protein form close binding interactions with Licochalcone D. (E) A detailed schematic representation of the interactions between Licochalcone D and ZNF131 residues. (F) The ligand torsion plot illustrates the conformational evolution of Licochalcone D for each rotatable bond (RB) throughout the simulation trajectory. The dial (or radial) plot depicts the torsional conformations observed during the simulation, and the bar graph summarizes the data, showing the probability density of the conformational twists

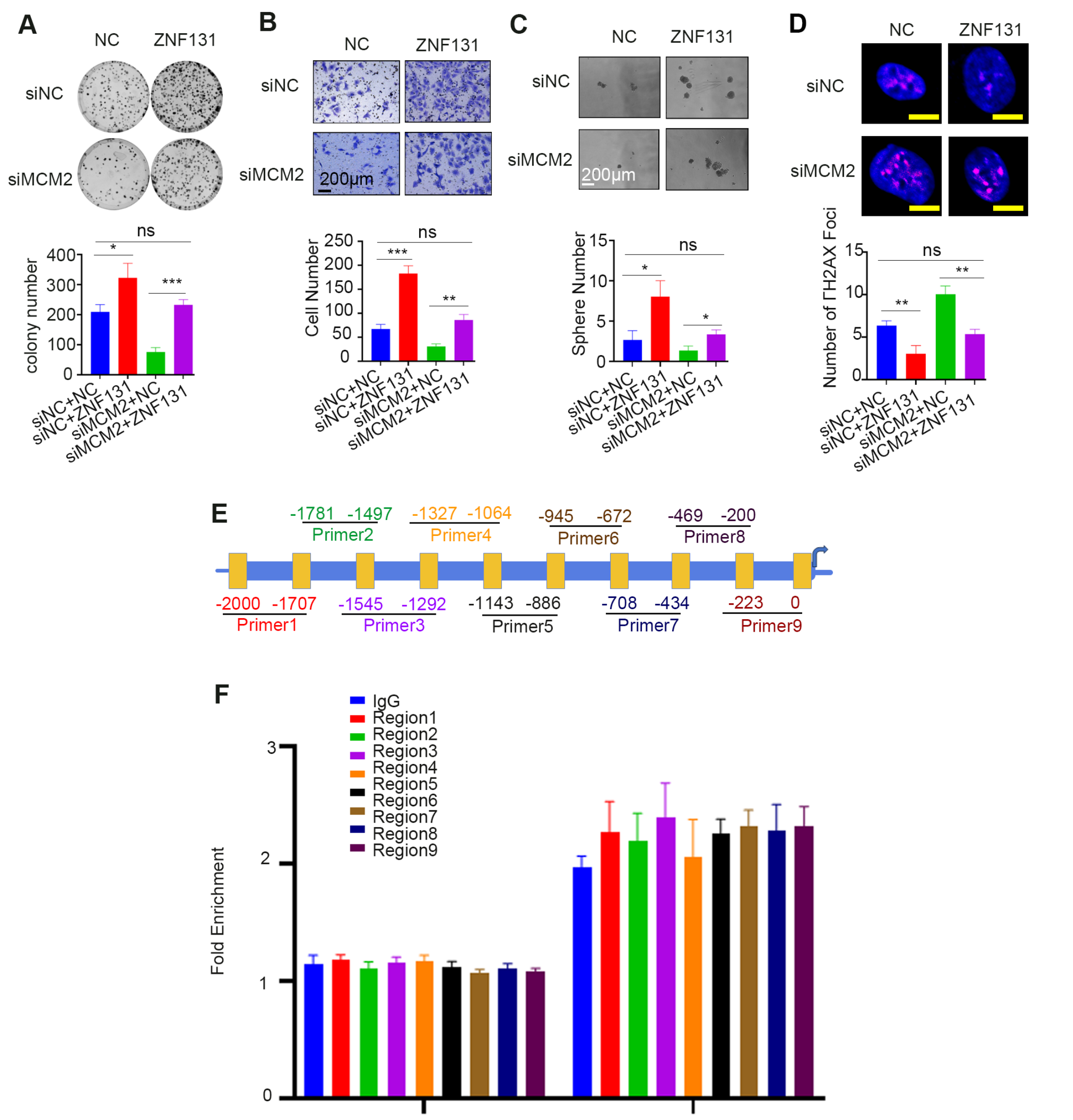

Figure S9. Knockdown of MCM2 Partially Rescued the Increased Proliferation, Invasion, Stemness, and DNA Damage Abrogation Induced by ZNF131 Overexpression

(A) Colony formation assays, (B) transwell assays, (C) sphere formation assays, and (D) immunofluorescence (IF) assays were performed to evaluate the effects on proliferation, stemness, invasion, and DNA damage in cells transfected with ZNF131-overexpressing plasmids, MCM2 siRNA, or control. Scale bar = 200 μm (for Sphere/Transwell assays) and 10 μm (for IF assays). (E) Diverse primers were designed to examine potential binding sites on the MCM2 promoter. (F) Additional ChIP

assays were conducted to identify the specific binding region of ZNF131 on the MCM2 promoter.

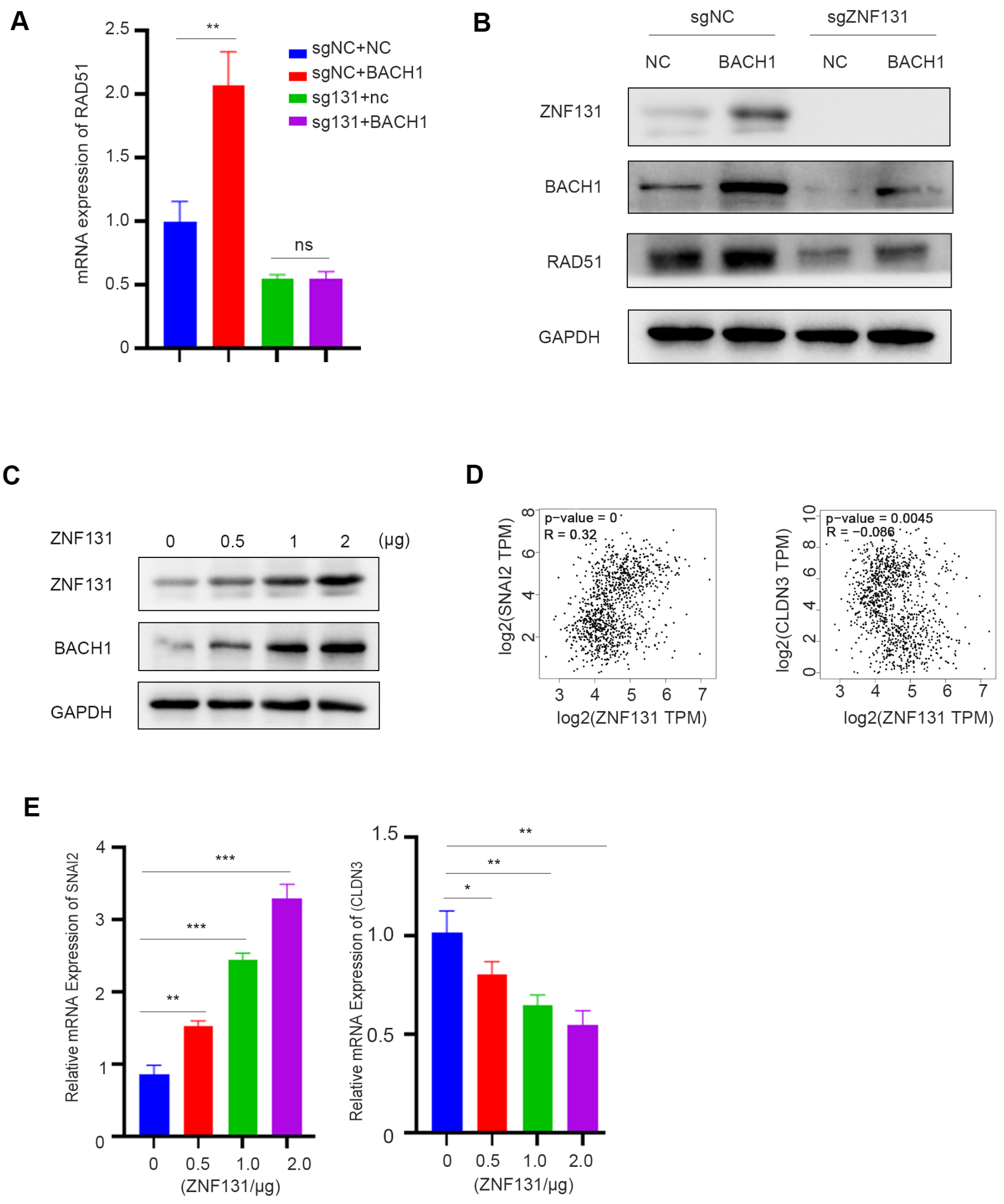

Figure S10. The Expression of BACH1 and Its Downstream Target Genes Is Modulated by ZNF131 in a Dose-Dependent Manner

(A-B) qPCR and Western blot (WB) assays were conducted to measure RAD51 mRNA and protein levels following ZNF131 overexpression combined with BACH1 siRNA in H1299 cells. (C) WB assay was used to assess BACH1 protein levels upon ZNF131 overexpression at increasing doses. (D) The GEPIA database was utilized to examine the correlation between ZNF131 and SNAI2 or CLDN3. (E)

qPCR assay was performed to measure SNAI2 and CLDN3 mRNA levels following ZNF131 overexpression at increasing doses in H1299 cells. Quantitative data are presented as mean ± SD from three independent experiments. Statistical significance was determined using t-tests (* $P < 0.05$, ** $P < 0.01$, *** $P < 0.001$).

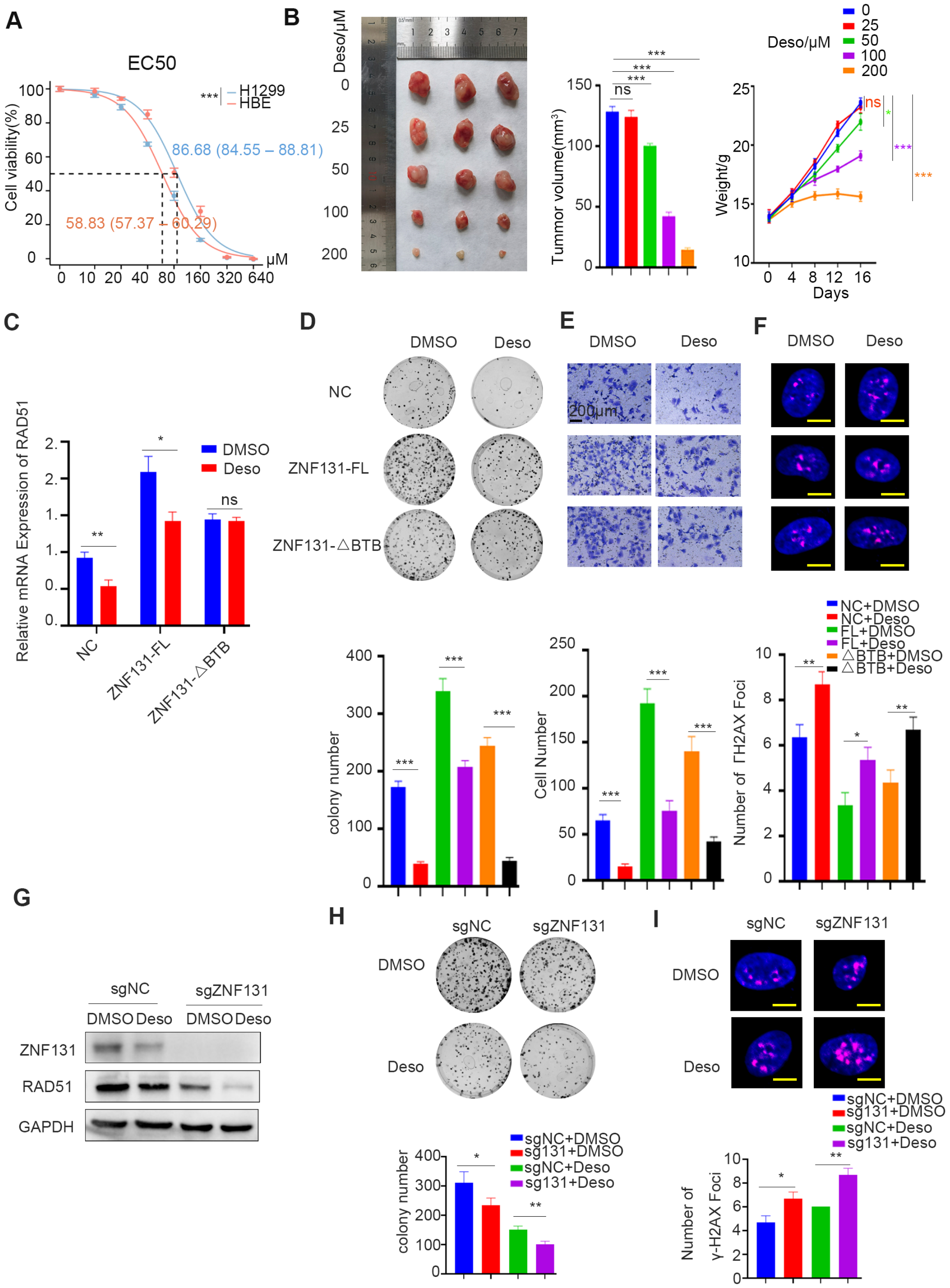

A
EC50
Cell viability(%)
H1299
HBE
86.68 (84.55 – 88.81)
58.83 (57.37 – 60.29)
μM
B
Deso/μM
Tummor volume(mm³)
Weight/g
Days
C
Relative mRNA Expression of RAD51
DMSO
Deso
NC
ZNF131-FL
ZNF131-△BTB
D
E
F
colony number
Cell Number
Number of ΓH2AX Foci
NC+DMSO
NC+Deso
FL+DMSO
FL+Deso
△BTB+DMSO
△BTB+Deso
G
sgNC
sgZNF131
ZNF131
RAD51
GAPDH
H
I
sgNC+DMSO
sg131+DMSO
sgNC+Deso
sg131+Deso
Number of γ-H2AX Foci

Figure S11. Deso Inhibited NSCLC Progression Partially by Targeting ZNF131

(A) Determination of IC50 values in H1299 and HBE cells overexpressing ZNF131 or control plasmids following treatment with Deso. (B) Xenograft assays were conducted to assess the effects of different doses of Deso treatment on tumor progression. (C) qPCR assays were performed to evaluate RAD51 mRNA expression in NSCLC cells treated with Deso, following overexpression of ZNF131-FL, ZNF131-ΔBTB, or control plasmids. (D-F) Colony formation (D), transwell (E), and immunofluorescence (IF) assays (F) were used to assess the effects on proliferation, invasion, and DNA damage in cells transfected with ZNF131-FL, ZNF131-ΔBTB, or control plasmids, respectively. (G) Western blot (WB) assays were conducted to evaluate RAD51 protein expression after Deso treatment in ZNF131-KO cells. (H-I) Colony formation (H) and IF assays (I) were conducted to assess the impact on proliferation and DNA damage in ZNF131-KO cells treated with Deso. Quantitative data are presented as mean ± SD from three independent experiments. Statistical significance was determined using t-tests (* $P < 0.05$, ** $P < 0.01$, *** $P < 0.001$).

Supplementary materials and methods

Immunohistochemistry

Samples were fixed in 10% neutral formalin, embedded in paraffin, and sliced in 4-μm thick sections. Immunostaining was performed by the streptavidin- peroxidase method. The sections were incubated with a monoclonal mouse anti-ZNF131 antibody (1:100; Sigma, St. Louis, MO, USA) or anti-RAD51 antibody (1：200; Proteintech, Chicago, IL, USA) at 4°C overnight, followed by biotinylated goat anti- mouse IgG secondary antibody. After washing, the sections were incubated with horseradish peroxidase-conjugated streptavidin–biotin (Ultrasensitive; MaiXin, Fuzhou, China) and developed using 3, 3-diaminobenzidine tetra- hydrochloride (MaiXin). Finally, samples were lightly counterstained with hematoxylin, dehydrated in alcohol, and mounted. Two investigators blinded to the clinical data semi-quantitatively scored the slides by evaluating the staining intensity and percentage of stained cells in representative areas.

Western blotting, Proteasome-inhibition and ubiquitination assays and immunoprecipitation

Total protein was extracted using a lysis buffer (Pierce, Rockford, IL, USA) and quantified with the Bradford method. Fifty μg of the total protein samples were separated by 10% SDS-PAGE, and transferred onto polyvinylidene fluoride membranes (PVDF; Millipore, Billerica, MA, USA). Membranes were incubated overnight at 4°C with the following primary antibodies: GAPDH and ZNF131 (mouse polyclonal, # H00007690-B0IP, Abnova), Myc- tag, DYKDDDDK Tag, RAD51, γ-H2AX (1:1000; Cell Signaling Technology, Danvers, MA, USA), BACH1 (1:1000, 14018-1-AP, Proteintech, Chicago, IL, USA), BACH1 (1:1000, sc-271211) and CUL-3(sc-166110)(Santa Cruz, CA, USA), GFP antibody (1:1000, Abcam, Cambridge, UK ab290). Membranes were washed and subsequently incubated with peroxidase- conjugated anti-mouse or anti-rabbit IgG (Santa Cruz Biotechnology) at 37 °C for 2h. Bound proteins were visualized using electrochemiluminescence (Pierce, Rockford, IL, USA) and detected with a bio-imaging system (DNR Bio-Imaging Systems, Jerusalem, Israel).

For proteasome-inhibition assays, cells were transfected with the indicated plasmids, and at 48-h post-transfection, the 26S proteasome inhibitor MG132 (s1748; Beyotime Biosciences) was added at a final concentration of 10 μM for 5 h, after which samples were collected. Cells were lysed in lysis buffer, and

cell debris was pelleted by centrifugation at 12,000 rpm for 10 min at 4 °C. Supernatants were collected for IP, which was performed using whole-cell lysates (~ 200 μg protein), 4 μg to 10 μg antibody, and 20 μL protein A/G agarose (P2012; Beyotime Biosciences). Cell lysates were precleared with 20 μL agarose A/G beads by rocking for 1h at 4 °C, after which beads were removed, and appropriate antibodies were added. Samples were then incubated with 20 μL agarose A/G beads by rocking for 4 h to 6h at 4 °C, followed by the addition of lysates and incubation with rocking overnight at 4 °C. Immune complexes were collected by centrifugation, followed by washing in cell lysis buffer before analysis (30103781).

For immunoprecipitation, a sufficient amount of antibody was added to 200 mg of protein and gently rotated overnight at 4℃. The immunocomplex was captured by adding 25 mL of protein A/G agarose beads (Beyotime, Jiangsu, China) and gently rotated for 3 hours at 4℃. Then, the mixture was centrifuged at 1500×g for 5 minutes at 4℃, and the supernatant was discarded. The precipitate was washed three times with ice-cold radioimmunoprecipitation assay buffer, resuspended in sample buffer, and boiled for 5 minutes to dissociate the immunocomplex from the beads. The supernatant was then collected by centrifugation and subjected to Western blot analysis.

The Ub-HA plasmid and the target plasmid were transfected into cells, and the 26S proteasome inhibitor MG132 (HY-13259, MedChemExpress, Monmouth Junction, NJ, USA) was added after a certain period of time with a final concentration of 20 μM. Subsequently, the immune complexes were collected for immunotrace analysis according to the experimental procedure of co-immunoprecipitation.

Immunofluorescence and EdU assay

Cells were fixed with 4% paraformaldehyde, blocked with 1% bovine serum albumin, and incubated overnight with Myc-tag (1:50), ZNF131 (1:50), BACH1(1:50), CUL-3(1:50) and γ-H2AX (1:50) at 4°C. Cells were then incubated with tetramethylrhodamine isothiocyanate-conjugated secondary antibodies (Cell Signaling Technology) at 37°C for 2 h. Cell nuclei were counterstained with 4′, 6-diamidino-2-phenylindole (DAPI). Epifluorescence microscopy was performed using an inverted Nikon TE300 microscope (Nikon Co., Ltd., Tokyo, Japan), and confocal microscopy was performed using a Radiance 2000 laser scanning confocal microscope (Carl Zeiss, Oberkochen, Germany). The cells were incubated with 20 uM EdU solution (Cellorlab, Shanghai, China) for 1 h, after overexpressing or knocking out

ZNF131.

MTT, colony formation assay

Cells were plated in 96-well plates in medium containing 10% fetal bovine serum at about 3000 cells per well 24 hours after transfection. For quantitation of cell viability, cultures were stained after 4 days by using the MTT assay. Briefly, 20 µl of 5 mg/ml MTT (Thiazolyl blue) solution was added to each well and incubated for 4 hours at 37 °C, then the media was removed from each well, and the resultant MTT formazan was solubilized in 150 µl of DMSO. The results were quantitated spectrophotometrically by using a test wavelength of 490 nm, each carried out in triplicate.

The A549 and H1299 cells were transfected with pCMV6 or pCMV6- ZNF131 plasmids, while the H358 and LK2 cells were transfected with negative control or ZNF131-sgRNA for 48 hours. Thereafter, six-well plates were inoculated with 1000 cells per well and incubated in a cell incubator for 10–14 days. Plates were washed with phosphate buffer saline and stained with Giemsa. The number of colonies with more than 50 cells was counted. The colonies were manually counted by microscope, each carried out in triplicate.

Luciferase reporter assay

Lipofectamine 3000 was used for transfection according to the manufacturer's instructions. Cells were plated in 24-well plates for 24h prior to transfection with pGL3-Basic Vector or pGL3-RAD51 (0.5 mg) reporter gene plasmids, together with the control plasmid pRL-TK (50 ng). After incubation for 30 h at 37.5℃, reporter gene expression was detected by the Dual-Luciferase Assay System (Promega, Madison, WI). ZNF131-mediated gene transcription activity was determined by the ratio of pGL3- Basic Vector to pGL3- RAD51 luciferase activity, which was normalized to Renilla luciferase activity from the control plasmid pRL-TK. All experiments were performed, in duplicate, a minimum of three times. To analyze the effect of BACH on ZNF131-RAD51 signaling, ZNF131 and BACH1 including their splicesome (0.5 mg) was cotransfected with pGL3-Basic Vector or pGL3-RAD51 for luciferase assays. Empty pCMV6 vector was added to control for the amount of plasmid DNA. Luciferase activity was measured in cellular extracts using a dual-luciferase reporter gene assay kit (Promega, San Luis Obispo, CA, USA). Each experiment

was repeated in triplicate.

Transswell assay

Treated lung cancer cells (2 x 10^5 cells per 200 μL) were inoculated into Transwells (8-um pore size, Corning, inc., Corning, NY, USA) for 24h to observe their migratory capacity. Cells that crossed the Transwell membrane were stained with crystal violet. Cells were manually counted by microscope, each carried out in triplicate.
